# Obesity and Lifestyle Drift: Framing Analysis of Calorie Menu Labelling in England in News Media

**DOI:** 10.34172/ijhpm.8649

**Published:** 2025-04-28

**Authors:** Nancy Karreman, Michael Essman, Benjamin Hawkins, Jean Adams, Martin White

**Affiliations:** MRC Epidemiology Unit, University of Cambridge, Cambridge, UK.

**Keywords:** Media Framing, Obesity Policy, Public Health, Individualism, UK

## Abstract

**Background:**

Successive government public health strategies in England have described structural influences of diet-related ill health, including obesity, while emphasising the solution of individual-level change in policy documents. This entrenchment of an individualistic policy paradigm, despite communicating a recognition of structural determinants of health on paper, has been termed "lifestyle drift." The 2020 government strategy, *Tackling Obesity*, included policies to address structural determinants of health like the physical and digital food environments but ultimately failed to shift responsibility for diet-related ill health onto structural factors. This study uses the contestation of calorie labelling (CL) in the out-of-home (OOH) sector, one of the strategy’s only two implemented measures, in English newspapers to investigate how the policy is framed, and the potential role of media framing in facilitating lifestyle drift.

**Methods:**

We systematically searched the Factiva database for articles from 12 UK national newspapers that discussed CL between January 2017 and May 2022, and assessed them relative to inclusion criteria. We then used a combination of reflexive thematic analysis (RTA) and framing theory to qualitatively analyse the framing of policy problems and the solutions meant to address them.

**Results:**

A total of 177 articles met our criteria. We found that media framing often reinforced individualism, personal responsibility, and moralisation of behaviour. It also emphasised perceived mixed and inconclusive evidence of CL’s effectiveness, unfairness to businesses, and unintended consequences, including negative impacts on the economy and people living with eating disorders.

**Conclusion:**

Despite an initial shift towards framing interventions to address obesity through a structural lens in *Tackling Obesity*, CL legislation and accompanying news coverage reflected a drift back towards individualism. To enact effective, structural change to address diet-related public health issues, policy discourses and approaches need to move away from individualising and moralising framing of both public health problems and potential solutions.

## Background

**Key Messages**

**Implications for policy makers**

- Framing is an important part of the agenda setting process, can affect the content of implemented policies, and can serve as a vehicle for industry influence on the policy-making process.
- Individualised framing of the problem of obesity and the policy solutions meant to address it detracts from efforts to address structural causes of ill health.
- Calorie labelling (CL) in England is a paradigmatic example of how framing can effectuate ‘lifestyle drift’ away from structural interventions.
- Policy-makers should resist lifestyle drift and ensure their communications reflect structural understanding of obesity and that this is reflected in proposed policy solutions.

**Implications for the public**

Successive obesity strategies in England have failed to achieve sustained reductions in obesity prevalence, in part due to too much focus on individual actions. We looked at how newspapers discussed calorie labelling (CL) on menus in England. Our findings show that media coverage often emphasises personal responsibility and relates individuals’ eating habits to their moral value, which can distract from more impactful solutions that target the root causes of poor health. To improve this, policy-makers and advocates should pay attention to how they discuss these issues, and the public should support shifting the focus away from blaming individuals.

For thirty years, UK government obesity strategies have emphasised individual behaviour change.^1,2^ The 2020 strategy for England, *Tackling Obesity*, was framed as addressing environmental influences.^3^ However, the strategies’ policy measures that target environmental factors, including advertising restrictions, have not so far been implemented.

*Framing* refers to the “interactive, intersubjective processes” by which we categorise and make sense of the world,^4^ both strategically and unconsciously in communication and reception.^5^ The framing of policy issues and solutions is an integral part of the policy-making process.^4,6^ Individuals use particular issue *frames* as interpretative lenses to understand the world around them.^7,8^ Actors frame issues tacitly to shape perceptions of social problems and their favoured policy responses.^6^ This “ideational” dimension of policy-making thus constitutes an important form of influence on policy formulation.^9,10^

 The predominant framing of successive UK government public health policies, including *Tackling Obesity,* has facilitated “lifestyle drift,” whereby initial recognition of “upstream” determinants of health drifts back “downstream,” resulting in policies designed to target individual “lifestyles.”^2,11,12^ This is evident in the UK government’s embrace of nudge theory^13-15^ in *Tackling Obesity*’s stated goal of “empowering adults and children to live healthy lives.”^2,3^ Even when policies to target upstream determinants of ill health are formulated, they are discursively (re)framed to be about individuals and their (problematic) behaviours – a phenomenon termed “upstream individualism.”^2^

 The use of framing strategies by commercial actors to neutralise the threat of regulation is well-documented across the tobacco,^16,17^ alcohol,^18,19^ gambling,^20^ and food industries.^9,21,22^ These health-harming industries are a key component of the commercial determinants of health: “the systems, practices and pathways through which commercial actors drive health and equity.”^23^ They use framing strategies in an attempt to “secure preferential treatment and/or prevent, shape, circumvent or undermine public policies in ways that further [their commercial] interests.”^24^ These include positioning corporations as good social actors through the use of corporate social responsibility for reputation management^23^; undermining proponents of whole-population regulation; attributing responsibility for health harms to a minority of consumers; promoting self-regulation and individual-level intervention; and arguing that population policies are illegitimate, unjust, ineffective, and damage both society and the economy.^24^ Framing thus constitutes a key facet of businesses’ discursive power, which both shapes business interests and confers legitimacy to pursue them.^10^

 Media, including print news, television, radio, and online forms, play an important agenda-setting role in the policy process by indicating which issues the public and policy-makers should consider most salient, or “newsworthy,”^25^ and framing them as worthy of action.^26-28^ Media coverage can thus influence the policy agenda in favour of specific interests, including those of politicians, institutions, or industry.^29^ Research on media framing is, therefore, important to understanding the public health policy-making process.^29,30^

 In this article, we investigate the framing of calorie labelling (CL) in the out-of-home (OOH) sector in print media from pre- to post-implementation in English newspapers. CL was first proposed in 2018 and was subject to substantial debate, leading to various adaptations before adoption in 2022 (Table 1). Evidence for the efficacy of CL is mixed,^31,32^ and post-implementation evaluations have shown uneven compliance with legislative requirements, limited impact on consumer purchasing, and no change in the number of calories purchased or consumed.^33-35^ Despite this, CL has been touted as a testament to the government’s commitment to “tackle preventable disease conditions such as obesity.”^36^ Reactions to the policy by public health actors and commentators have been mixed^37^ and hotly debated in the media.^38^

**Table 1:** Policy Timeline of Calorie Labelling in England, 2018-2022

**Date**	**Event**
June 25, 2018	May government obesity strategy published
September 14, 2018–December 7, 2018	First consultation on CL (content)
September 5, 2018	Letter of opposition from Treasury
May 10, 2019	Proposal to restrict to businesses with >250 employees
July 27, 2020	Government response to first consultation on CL published
July 27, 2020	Johnson government obesity strategy published
July 30, 2020–September 9, 2020	Second consultation on CL (enforcement)
May 11, 2021	Alcohol dropped from proposed policy and scope restricted to outlets with >250 employees
April 6, 2022	CL legislation implemented in England

Abbreviation: CL, calorie labelling.

 We sought to identify the key arguments made about CL by diverse actors in newspaper articles in order to address our two-part research question: How is CL framed as a solution to the policy “problem” of obesity, and how is obesity reconstructed to fit the proposed solution of CL? We captured the language and rhetorical devices used to communicate actors’ perspectives, and the evidence cited in support of their positions. Though we did not limit our analysis to framing by commercial actors, this study contributes to the body of scholarship on corporate political activity and the commercial determinants of health by exploring links between media framing and the maintenance of an industry-favourable policy paradigm.

## Methods

###  Overview and Theoretical Approach

 NK systematically searched English editions of 12 UK national newspapers published May 2017–May 2022. These newspapers were chosen because they were archived within the Factiva database at the time of our search in May 2022 and accounted for approximately 4.5 million units sold in January 2020, with online access not reflected in these totals^[1]^. ^39^ After screening for inclusion, we performed an inductive, reflexive thematic analysis (RTA)^40^ of characterisations of policy problems and proposed solutions^6^ within included articles. We followed the protocol uploaded to Open Science Framework in July 2022.^41^

 We adopted a qualitative approach because it enabled us to engage with the nuance and complexity of media discourse, as well as aligning with our epistemology and research questions. We analysed articles through RTA informed by framing theory and a constructionist epistemology that recognises both policy problems and solutions as discursively constructed through framing as a process of problem definition. RTA is an adaptable approach to qualitative, text-based analysis^40^ and framing theory provides a useful framework for elaborating the internal logic of the (often contradictory) positions of actors engaged in policy debate.^18^ Which policy problems make it onto the policy agenda, in what form, with what urgency, and involving which actors is not a natural process but is the result of political contestation between policy actors with differing power.^42^ Our work is also informed by Van Hulst and Yanow,^4^ who extend the work of Rein and Schön^6^ and draw on Bacchi^43,44^ to argue that policy problems are framed through a process of *naming* or describing the issue through the use of metaphor; *selecting* what is (or is not) relevant to the issue at hand; and through *storytelling*, whereby new situations are described using familiar narrative patterns that are comprehensible to policy-makers and citizens.

###  Search Strategy

 After piloting, NK searched Factiva (Bloomberg) on May 24, 2022 with the stemmed keywords calori* and menu* and label* for articles published since May 3, 2017 in 12 UK national newspapers and their corresponding Sunday versions (Independent, The Guardian, Daily Mirror, The Sun, The Times, Daily Express, Financial Times, Daily Star, Sunday People, Morning Star, Daily Mail, and The Telegraph) in English. May 2017 was chosen as a starting point to create a five-year sampling frame until CL implementation in April 2022 and capture discussion of CL before its first inclusion in a government obesity strategy. Full search details are included in Table S1 (Supplementary file 1).

###  Screening: De-duplication

 The Factiva database automatically removed 178 duplicates (those classified as “identical”). A further 21 duplicates were automatically removed after being uploaded to Covidence (Clarivate). NK and ME then carried out manual de-duplication, following the strategy outlined by Yau et al.^45^ An article was deemed duplicate if the author and title were the same, or similar, and more than 80% of the content was the same.^45^ In these cases, the first article read (alphabetical by author name) was kept. This resulted in a further 15 articles being removed.

###  Screening: Inclusion Criteria

 NK and ME independently assessed all 436 remaining articles relative to inclusion and exclusion criteria outlined in Table 2. Disagreements were resolved through discussion.

**Table 2:** Inclusion and Exclusion Criteria for Newspaper Articles

**Inclusion Criteria**	**Exclusion Criteria**
Includes mention of CL in OOH sector in the body of the text Published between May 2017 and May 24, 2022 All article forms, including letters to the editor, except news-in-briefs and roundups	Does not include mention of CL in OOH sector in the body of the text Only discusses CL in a context other than England (eg, USA, Scotland) News-in-briefs or roundups

Abbreviations: CL, calorie labelling; OOH, out-of-home.

 The screening process resulted in 177 articles being included (full details in Table S2 of Supplementary file 2). The full screening process and results are depicted in Figure.

**Figure:**
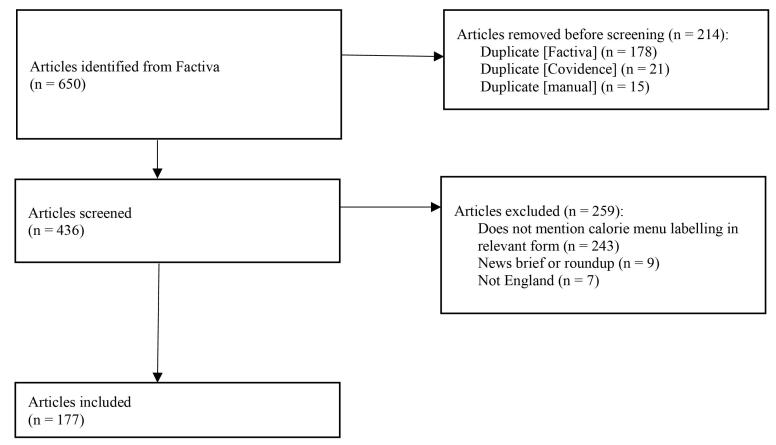
Diagram Depicting the Search and Screening Strategy of Newspaper Articles.

###  Data Extraction and Preliminary Analysis

 NK extracted data from included articles using a standardised form in Covidence. Information extracted included: article title, author(s), publication date, and newspaper. We also incorporated preliminary analysis questions in the extraction form, including:

- What is the problem framing?
- What is the solution framing?
- To what degree does this article focus on calorie menu labelling?

These sensitising questions facilitated our familiarisation process and served as a starting point for iterative movement between texts, data extraction tools, and analysis. The final question was used to prioritise articles for main analysis and created five groups: articles that had CL as their *primary* focus, articles that had it as a *major* focus, articles in which it was a *minor* focus, articles that only *mentioned* it, and articles that focussed primarily on a *related topic*, like traffic light labelling (Table 3). We decided to maintain articles in the last category because these also included framing of CL. All articles were ultimately included in our analysis and these categories served no further role in the interpretation or presentation of our results. NK then imported all 177 included articles into NVivo 12 Pro software (Lumivero) for full qualitative analysis.

**Table 3:** Classification of Included Articles by Degree of Focus on Calorie Labelling

**Degree of Focus on CL**	**Number of Articles**
Primary focus (most)	91
Major focus	24
Minor focus	29
Mention only (least)	26
Related topic (eg, traffic light labelling)	7
Total	177

Abbreviation: CL, calorie labelling.

###  Main Analysis

 We first used RTA to inductively code articles and generate themes, then organised themes into problem and solution framings. NK led analysis of each included article via RTA which, as described by Braun and Clarke,^40^ is a form of qualitative analysis that involves the generation of codes and themes from patterns within a dataset. Reflexivity explicitly recognises the role of the researcher in creating meaning from data and requires critical interrogation of how researchers’ characteristics, experience, and knowledge influence interpretation.^46^ A reflexivity statement is attached in Supplementary file 3. We also drew on the recently published Reflexive Thematic Analysis Reporting Guidelines in our writing and editing process.^47^

 Analysis of articles included six phases: familiarisation; coding; generating initial themes; developing and reviewing themes; refining, defining, and naming themes; and writing up.^40^ NK developed a preliminary coding framework based on analysis of a random sample of 62 articles (approximately one third of total included articles). This framework was then applied to all other articles in order of their degree of focus on CL, from greatest to least, and was adapted iteratively throughout our analysis (Table 3). NK prepared preliminary themes and refined them through discussion with the rest of the research team. Themes were then organised into problem and solution framings. How actors were framed within these was noted and used to contextualise quotations.^6^ All framings developed are reported below.

 We present our findings in two sections: framing the policy problem and framing the policy solution. Within each section, our findings are structured by subheadings that highlight common themes between instances of framing, while the framings included below each subheading correspond to the level of codes. This represents framing at two distinct levels of granularity (themes and codes).

## Results

 Our analysis developed multiple framings that promoted and opposed implementation of CL. These are categorised by whether they framed the policy problem or solution in Table 4. It is important to note, however, that the division between problem and solution framings is imprecise and accommodates framings that span both heuristic categories; it should be interpreted as a constructed analytic tool rather than a natural category. Similarly, framings may overlap and the same portion of text may communicate several framings at once.

**Table 4:** Problem and Solution Framings

**Problem Framings**	**Solution Framings**
Obesity is a pressing and important issue that should be addressed by government intervention. Obesity is a crisis Obesity is caused by increased consumption of unhealthy OOH food Consumers lack information to make good choices in the OOH environment This legislation will not address the “real” causes of obesity. Obesity is caused by factors that CL will not address (structural factors, poor lifestyle choices) Obesity is not the only problem (eating disorders are the real problem)	Government intervention is needed because individuals on their own will not be able to change the food environment. CL will empower responsible consumers CL will encourage reformulation Making healthy choices should be a matter of personal responsibility, not government intervention. CL will be ineffective CL is unnecessary CL will be unfair CL will ruin food culture CL is a slippery slope towards over-regulation We should instead target other structural determinants of ill health. CL will be ineffective CL will cause mental health harm

Abbreviations: CL, calorie labelling; OOH, out-of-home.

###  Framing the Policy Problem

 Obesity functioned as a key point of reference in policy debate, to which CL was considered as (part of) the solution, with problem framings diagnosing mechanisms responsible for obesity’s production (Table 4). Almost all problem framings referenced the scope, severity, or implications of the rising number of people living with obesity in the UK. However, how the problem of obesity was defined differed depending on who was framed as responsible for its origins: as an issue caused by individuals’ behaviours, or by an unhealthy OOH environment. Some of these framings were common across policy attitudes (ie, pro- or anti-CL) while others were specific to particular communities and stances towards CL, which reflects the policy’s inclusion in various strategies.^3,48^

####  Obesity Is a Crisis

 Framings that sought to demonstrate the scale, severity, and urgency of the problem of obesity spanned both anti- and pro-CL positions. Population statistics describing the prevalence of obesity (“Almost two-thirds [63%] of adults in England are overweight or obese and one in three children leaves primary school too fat”^49^); reiterating its costs to the National Health Service (NHS) (“the NHS spends £6.1 billion per year on overweight and obesity-related conditions”^50^); emphasising the negative associations between excess weight and severity of COVID-19 infection (“obesity also increases the risk of dying from coronavirus”^51^); and framing obesity as a national embarrassment (“We are a nation of fatties”^52^) were employed to emphasise the scale and severity of the problem.

 It is also important to note the repeated use of crisis language to describe obesity. Government sources called it “a time bomb”^53^ and said that it was “combatting” obesity.^54^ Media coverage similarly cast public health measures as “part of the Government’s wider war on obesity.”^55^ However, as pointed out by a dietician interviewed by *The Independent*: “using terms like ‘battle against obesity’ is dangerous territory as it can be misunderstood that you’re either on the ‘good or bad side.’”^56^ This framing also had the effect of problematising the bodies of people living with obesity and their dietary practices, rather than highlighting the environmental, social, economic, and political conditions that produce obesity. Framings that attributed moral qualities to individuals and groups were used by actors on both sides of CL policy debate and are elaborated further below.

####  Obesity Is Caused by Overconsumption of Unhealthy Out-of-Home Food

 One prominent framing explained the problem in terms of increased consumption of “unhealthy” food and an information asymmetry between consumers and businesses in the OOH environment. Both pro- and anti-CL media reporting attributed increasing obesity rates to increased OOH consumption: “eating out had become ‘the norm.’”^57^ This built on a general claim that Britons “significantly underestimate”^58^ the number of calories in food that they consume by 200-500 kcal per day.^59-61^ OOH food was also identified as especially calorific,^62,63^ less healthy than home-prepared food,^64^ and “over-running our high streets.”^65^ Concerns about the routinisation of eating outside of home, as opposed as to as “just a treat,”^63^ accompanied concerns that the OOH environment was an “information-free zone”^66^ to strengthen the need for calorie information.

####  Consumers Lack Information to Make “Good” Choices in the Out-of-Home Environment

 When announcing CL in 2018, then-Public Health Minister Steve Brine said: “Families want to know what they are eating when on the go, but in many cafés, restaurants and takeaways this information is not available.”^38^ Lack of information was framed as bad because of its putative effects on decision-making and because it contravened customer preference, a paramount principle of the free market supply and demand model. Brine continued: “we have a right to know the nutritional content of the food we give to our children.”^38^ The consumer right to information, especially with regards to children and families, was thus also marshalled to support the need for CL.

 Pro-CL coverage also framed the policy as being in the interest of industry. Large chains where labelling already existed, including café chain Starbucks and fast-food restaurant McDonald’s, were touted as early adopters of voluntary CL.^67^ This attempt by government and public health advocates to articulate a shared interest with the food industry was mirrored by businesses. In an article covering online delivery platform Deliveroo’s pre-emptive introduction of CL, a company spokesperson told *The Daily Mail* that “the move to publish calories was partly in response to demand from customers.”^68^ However, CL opponents also claimed to be following consumer preferences. An owner of a fish and chips shop described his role as a purveyor of less-healthy food options as “a public service” in response to consumer demand.^57^

####  Obesity Is Caused by Factors That Calorie Labelling Will not Address

 However, locating the problem of obesity in a deficit of calorie information was fiercely contested. CL opponents argued that calories were not the root cause of obesity. Instead, the root cause of the issue was attributed to the “obesogenic environment”^69^; the immorality of “obese people” who “don’t care how many calories they eat”^70^; or cultural norms.^71^

 Restaurants also drew on the expertise of in-house dieticians and nutritionists to claim that CL was overly simplistic and did not relate to real nutritional value^72^:

 Pho’s [Vietnamese restaurant chain] nutritionist […] says: […] “*A number displayed on a menu certainly does not dictate how healthy you are or the quality of your nutrition.*”

 Obesity was also framed as “too complex” to be resolved by CL^51^ or already resolving itself, as demonstrated by plateauing obesity rates.^73,74^

 These framings differ in terms of who they hold responsible for causing obesity: asserting that obesity is caused by structural factors (eg, the economy, politics, the environment) tends to redirect focus away from the individual and their behaviours while framing obesity as the result of “lifestyle choices” tends to problematise the consumer and their actions.

####  Obesity Is not the Only Issue

 Newspaper coverage also emphasised the increased numbers of people living with eating disorders, particularly during the COVID-19 pandemic.^75^ In contrast to people living with obesity, people living with eating disorders were framed as vulnerable and in need of protection. This juxtaposition at the heart of CL discourse was reflected upon by an interviewee who self-identified as overweight in *The Independent*^76^:

 “*If I was anorexic, people would feel sorry for me. If I was pulling my hair out because of an obsession or was self-harming in other ways, people wouldn’t say, ‘just stop doing it.’ It seems acceptable still to jeer at fat people. […] You should feel guilty about being fat.”*

 This framing opposed CL by asserting that the problem needed to be expanded to accommodate concerns about eating disorders. Actors also critiqued the solution of CL for being an inappropriate answer to the problem of obesity. These framings are investigated in the next section on solution framing.

###  Framing the Policy Solution

 Solution framing refers to the use of rhetorical framing to characterise the content, purpose, and effectiveness of policy solutions, with particular policy solutions compatible with promoted problem framings, while others are precluded. This section elaborates seven framings of CL, with two in favour of the policy and five against it.

####  Calorie Labelling Will Empower Responsible Consumers 

 CL, as an information-providing intervention, was framed as grounded in a rational choice-based mechanism in which providing relevant information was perceived to enable consumers to make more informed, and therefore healthier, “choices.” For example, then-Public Health Minister Maggie Throup said: “It is crucial that we all have access to the information we need to maintain a healthier weight and this starts with knowing how calorific our food is.”^77^

 Though some cross-sector consensus between grocery retailers and OOH businesses about providing information to consumers existed, who was responsible for resolving this information gap was contested. For example, a Pho representative said it was “not the place of restaurants to educate customers on […] nutrition.”^72^ It disavowed responsibility for substantive education on how to use calorie information.

 Health advocates framed calorie information as helpful to groups trying to manage their calorie intake. For example, a spokesperson for Diabetes UK said that people living with diabetes desired calorie information to help them manage their condition.^78^ The organisation also commissioned a poll that was cited as evidence that consumers wanted more informational labelling in restaurant settings.^79^ Media commentators speculated that people who were trying to lose weight would also find calorie information helpful.^80^ This framing promoted CL as being in the public interest, or at least that of specific groups.

 Consumers were also framed as responsible for their health-related decisions. Former Foreign Secretary Dominic Raab said “we all need to take more personal responsibility” for the health of the nation.^81^ Reporting on then-Prime Minister Boris Johnson’s contradictory “damascene conversion”^82,83^ from an anti-nanny state libertarian to weight loss advocate through contracting COVID-19 characterised him as an exemplar of personal responsibility.^84^ An oft-cited Public Health England survey that found that the majority of respondents believed that responsibility for health outcomes lay with individuals ahead of government or industry reinforced this personal responsibility framing.^85^

 However, the consumer was also expected to indulge in “treats” when eating outside of home.^49,72,78,86-89^ As one commentator said: “Eat healthily most of the time, treat yourself now and again and exercise.”^90^ Individuals were attributed two seemingly incommensurable traits: responsibility and indulgence. People who were lucky to be able to consume in this way and maintain a ‘healthy’ weight were idealised. People who failed to do so were framed as stupid, lazy, and greedy. In *The Independent*, one interviewee characterised the Johnson government’s obesity strategy as stigmatising and hypocritical^76^:

 “*It’s the same spiel and adds to the age-old stereotype of obese people; they’re uneducated, lazy slobs who eat too many takeaways because they’re greedy. It points the finger at ‘fixing’ already obese people rather than preventing obesity.” *

 By providing information and framing consumers as responsible for implementing it, businesses and the government offloaded responsibility for the well-being and financial costs of obesity onto individuals while enabling industry promotion of (over)consumption of less healthy food options through policy inaction.

####  Calorie Labelling Will Encourage Reformulation

 Government also framed product reformulation as a potential mechanism of impact of CL. In media coverage of its press release announcing the policy’s implementation in April 2022,^91,92^ the government listed reformulation as a subsidiary goal of CL^93^:

 “*The legislation […] aims to ensure people can make more informed, healthier choices when it comes to eating food out or ordering takeaways. Displaying calorie information may also encourage businesses to provide lower calorie options for their customers.” *

 This language mirrored the framing of CL as “encouraging” (but not requiring) individuals to change their behaviour. Media coverage tied this reformulation framing to a cross-sectional study that found that items from UK restaurants with CL had 45% less fat and 60% less salt.^94^ The study was framed as demonstrating that restaurants could be “shamed” into changing their product offer.^64^ Most mentions of reformulation came after this study was published, and indeed after CL had already been adopted onto the policy agenda.

####  Calorie Labelling Will Be Ineffective

 Evidence of the effects of CL from supermarket settings was used to suggest that CL would be similarly effective in the OOH sector.^77^ However, when obesity causation was framed as environmental or structural (eg, the influence of commercial marketing or economic inequality), a policy like CL that focussed on individual choice appeared less appropriate. This objection was epitomised by the then-Director of Obesity UK^77^:

 “*‘The reality is there’s so many different factors that contribute to obesity,’ […] it would be ‘better to focus attention on restricting the availability of food, rather than placing the emphasis solely on individuals to make choices.’” *

 Similarly, some commentators argued that what was really needed to “solve” obesity was the elimination of structural barriers to healthier food^76^ and addressing poverty.^95^ However, other alternatives were distinctly individualistic, including increasing exercise and physical activity,^74,96^ better parenting,^70^ and education.^72,91,97,98^

 The public was framed as either not caring about calories (and by extension, their weight and health), or otherwise unable to take action on calorie information.^99^ This framing alleged that, in particular, less socio-economically privileged people were either unwilling or unable to change their behaviour^99^:

 “*Those in our society most prone to obesity - who are mainly to be found in less well-off and poorly educated households - are already the least likely to take any notice of nutritional advice. They are also more likely to eat the junk food and sugar laden drinks to be found at the likes of McDonald’s, where calorie labelling shows no sign of slowing custom.” *

 Research evidence of the efficacy of CL was also framed as minimal, poor quality, or non-existent, in contrast to evidence opposing its implementation. A spokesperson for eating disorder charity Beat said^49^:

 “*We know […] that including calories on menus can contribute to harmful eating disorder thoughts and behaviours worsening. […] There is also very limited evidence that [CL] will lead to changed eating habits among the general population.”*

 Alongside presenting evidence from Beat’s surveys and call centre, opposition to CL re-framed findings from a 2018 Cochrane review that CL had the potential to reduce calorie consumption^31^ as “just 12 per cent”^100,101^ and “hardly a whopping saving,”^73^ and emphasised that its findings were based on “a small body of low-quality evidence.”^97^

####  Calorie Labelling Is Unnecessary

 CL was framed as unnecessary because people already know what is good for them and, contradictorily, that businesses’ voluntary initiatives to reduce calories are already working.

 Opponents emphasised customer intuition and existing knowledge to deny the need for additional information. As a spokesperson for free-market think tank The Adam Smith Institute argued: “We don’t need government enforced calorie counts to tell us something we already know.”^102^ Similarly, a Treasury official suggested that the department opposed CL because people already “know a burger is fattening.”^103^ This was not, however, a narrative restricted to solely free market-aligned actors. In *The Independent*, an interviewee who self-identified as overweight said that calorie information was unnecessary because people living with obesity were already subjected to the social normativity of dieting, thinness, and fat-shaming.^76^

 On the other hand, when calorie overconsumption was acknowledged as a real issue, industry actors argued that voluntary initiatives to cut calorie content were already working, rendering mandatory legislation unnecessary. As the Portman Group, an alcohol industry-funded social aspects organisation^104^ argued: “We are committed to working in partnership with the Government. […] We have shown time and time again that the voluntary approach works.”^102,105^ The Treasury also demonstrated a strong commitment to industry partnership leading to CL being dropped from the policy agenda in 2018,^106^ and restricted to businesses with more than 250 employees when it was revived two years later (Table 1).^107^

####  Calorie Labelling Is Unfair

 The allegedly unfair burden that CL would impose on industry, and particularly small businesses, served as a powerful framing seeking to limit the scope of regulation. Costs to industry featured more prominently than the benefits of the policy in media coverage. While the estimated £7mn annual cost to industry^[2]^ was commonly cited, only one article referenced the social benefit of policy implementation,^108^ estimated at £5.5bn over 25 years^[3]^. ^109^ Similarly, it was claimed that businesses could pass costs onto consumers through increasing prices, reducing product ranges, or employing fewer staff.^84,110-112^

 The costs of CL were framed as particularly unfair in the context of Brexit and the COVID-19 pandemic. As the British Beer and Pub Association (BBPA) noted^113^:

 “*After more than a year of being forced to close fully or operate under severe loss-making restrictions, now is not the time to heap burdensome and expensive regulation on our pubs. […] Calorie labelling would be kicking pubs and brewers when they are down.” *

 That costs would impact smaller pubs harder was framed as discrimination^114^ and “unBritish”^82,102^ given the apparently integral role of pubs in British society.

 Some commentators, including small business owners, argued that the legislation should apply only to large chains because they were largely responsible for the availability of less healthy food options. *The Telegraph* reported that support for CL from larger firms might be part of a cynical strategy to “nobble the competition” from smaller businesses.^115^

 CL was also framed as being unfair to a responsible majority whose eating experience should not be “ruined” due to the failings of an irresponsible minority. This framing interestingly contrasts with that of obesity as a “crisis.” People living with obesity were framed as lacking the appropriate willpower or respect for their fellow consumers to take responsibility for their own health, and therefore were at fault for both the obesity crisis and CL. Parents were singled out as irresponsible for failing to parent “correctly”: “The sort of families whose children tend to get too fat are most unlikely to be swayed by figures stuck on menus […] If parents cannot enforce such restraints, it is hard to see how governments can.”^116^ In the effort to provide information “so parents can make informed choices about what their families eat,”^117^ failure to use information properly was framed as irresponsible and, when caring for children, immoral. A lack of personal responsibility could not, and should not, be solved by government intervention, and therefore CL was framed as both inappropriate and ineffective.

####  Calorie Labelling Will Ruin Food Culture

 CL was framed as having important unintended negative consequences on British food culture. Dining out was framed as “enjoyment and pleasure”^101^ and indulgence was constructed as an inherent part of British food culture.^97^ Seeing calorie labels on menus, it was argued, would undermine diners’ enjoyment of their meals.^118^ *The Telegraph* captured this sentiment with the headline: “You can count on menu calorie labelling to spoil all the fun.”^119^ Similarly, the BBPA commented that most customers “just want to come in, have a treat and not feel guilty about it.”^72^

 This “ignorance is bliss” framing was contested by public health actors: “Having a takeaway or eating out is no longer a treat, it’s a regular part of everyday life, yet too often menus are information-free zones.”^66^ To reconcile this, framings opposing CL emphasised the occasionality of OOH consumption in order to reconfigure indulgence as part of a healthy lifestyle. For example, a spokesperson for Papa John’s pizza chain described their menus as containing “treat” options that consumers could occasionally consume as part of a “balanced diet.”^49^

 In *The Times*, government was evocatively constructed as the uninvited enemy of fun on a night out and antithetical to indulgent, treat-based food culture^120^:

 “*[As] the menus arrive that I realise there are actually now three people on this date. Me, the missus and …who’s that nosy, unsmiling git in the corner? That’s the government.” *

 This was echoed by a chef opposed to CL who argued that^91^:

 “*[We’ll] lose the love of food[,] we’ll lose an idea of nutrition and deliciousness and what we should be eating and it will just be a main focus on a number[,] and I just think that’s […] dangerous.”*

####  Calorie Labelling Will Cause Mental Health Harm

 Enjoyment of food and awareness of its potential health impact were constructed as directly antithetical. The BBPA objected to CL as poorly timed for pubs and mental well-being after COVID-19 lockdowns^113^:

 “*The British people have had months without going to their local. It has been tough mentally and socially. Let them enjoy it again […] the pub has an important role to play in tackling loneliness and improving mental health.” *

 Dining out was discussed by campaigners as a “refuge” from calorie information^75,97^ for people living with eating disorders that was threatened by CL. Opponents of CL countered pro-policy, informed-choice framing by reframing calories as inherently associated with calorie counting and restriction, and therefore harmful. In a 2018 letter to *The Telegraph*, eating disorder charities and Members of Parliament wrote that they believed CL would be both ineffective in addressing obesity and damaging to those living with eating disorders.^98^

 A Beat campaigner framed CL as sacrificing the well-being of people living with eating disorders for the sake of combatting obesity:

 “*The only thing that’s certain about calorie labelling is that it will threaten the lives of people with eating disorders […] The Government is willing for the lives of people with eating disorders to be collateral damage in their fight against obesity.”*^121^

 Although CL legislation required businesses to have menus without calorie labels available on request,^93^ this still put the onus to navigate the food environment, and its impacts on well-being, on the individual.

####  Calorie Labelling Is a Slippery Slope

 Framing CL as a “slippery slope” towards authoritarianism was used to undermine support for the policy regardless of evidential claims. A belief that government regulation is ineffective and may have unintended consequences pervaded this discourse in the form of the “nanny-state” objection. This rhetorical device was used to object to the perceived unacceptable intrusion of the state on individual liberty, and the substitution of its judgement for that of the individual.^82,113,114^

 The nanny-state framing was used to allege that in making public health legislation like CL, the government treats adults in an “infantilising” manner^122^ and patronisingly polices their behaviour with “finger wagging.”^113^ Public health advocates were framed as “puritans”^74^ who are “excessively bossy”^123^ and “interventionist.”^81^ It was also associated with a populist rejection of a condescending elite:

 “It’s also another stick with which to beat the working classes as, obviously, high-end, Michelin-starred gaffs won’t need to show the calorie hit of their nitro-poached mousse palate-cleanser.”^118^

 This sentiment notably contradicts the way that people living with obesity, and especially those from lower socioeconomic backgrounds, were framed elsewhere as unintelligent and irresponsible.

 In this framing, consumers already know what’s good for them and choose to consume accordingly; it creates a moral hazard to substitute the judgement of the state for that of individuals. Even though CL provided information to consumers to facilitate their choices, because it was implemented by the state, it was seen as an inappropriate intervention into individual liberty.

## Discussion

 We investigated the framing of CL throughout the five-year period leading up to and including implementation, tracking framing strategies and the understandings of the public, public health actors, businesses, and the state that they promoted in news media. The greater focus on framings that generally opposed CL reflects the spread and diversity of framings in data we analysed. Pro-CL media coverage was focussed on responsible, informed choice and reformulation, while anti-CL framings were more diverse, and this is reflected in our presentation of our findings.

 CL was framed as providing information, both to influence consumers to make “informed choices” and induce businesses to reformulate their products. Through an “empowerment” framing, consumers were held responsible for the outcomes of their dietary “choices.” Anti-CL framing argued that CL would be ineffective, and either voluntary and educational initiatives or structural changes (eg, addressing poverty) would be more appropriate to address obesity. Evidence was framed strategically to provide support for whichever policy position was advanced, at times regardless of quality or evidential standard. Opponents framed labelling as unfair, unnecessary, and as an inappropriate solution to obesity. Though CL supporters framed the policy as helpful to prevent obesity and enable weight management, opponents framed it as having negative unintended consequences on British food culture and on people living with eating disorders. British identity was related to strong self-determination, through informed choice, as well as the ability to self-regulate indulgence in “treats” in the OOH sector; CL was portrayed as a patronising, nanny state measure that unnecessarily policed the choices of adults. Finally, CL and public health advocates were characterised as the “nanny state” and a moral hazard by anti-CL coverage.

###  Interpretation

 This analysis of the discursive framing of CL illustrates the complex and ideologically charged nature of political debate. The informed choice model that underpinned CL assumed both that consumers considered health to be an important outcome and that they had the capacity to act in accordance with these preferences. However, this mechanism was not usually made explicit in news articles, including those in support of the policy. The elision of information provision into healthy decision-making mirrors how obesity is used as a proxy for health^124^ and allowed policy proponents to leave unaddressed *how* information translates into healthier “choices” and better population outcomes. Similarly, when proposing alternatives to CL, its opponents struggled to differentiate between information provision and education, reflecting a desire for individuals to become “educated” rather than explaining how education programmes would address obesity.

 Debates about CL were marked by several contradictions: education and information-based approaches were best, but should not be mandated through legislation; consumers should (over)indulge but do so responsibly or face moral condemnation; and the “nanny state” constituted a form of class control, yet less socioeconomically privileged people living with obesity were disparaged as too unintelligent, unmotivated, or selfish to take action for their own health. The rhetorical deployment of “balanced consumption” is striking for its similarity to the alcohol industry’s attempts to frame alcohol consumption as part of a normal, healthy “lifestyle.”^125,126^ Similarly, though both restriction and overconsumption can be hallmarks of disordered eating, disorders associated with restriction were not perceived as the result of individual failings in the way that obesity was. The framing of these two archetypes as opposites in CL coverage also denied potential overlap between people living with obesity and those living with eating disorders.^127^ Using combative and morally divisive language stigmatises people living with obesity, causes moral panic, and increases health inequalities.^128,129^

 These findings also speak to the political framing of evidence to advance support or opposition regardless of “the facts.”^130^ For example, experts asserted that obesity was complex and the result of biological, genetic and social factors rather than solely personal “choices,” this was reinterpreted in media to signify that CL would not “work” because it failed to address the totality of obesity causation.^55^ These frames parallel well-documented industry strategies to deny the efficacy of or need for legislative interventions in the face of complexity.^24,134,135^ Evidence also functions rhetorically to legitimate a particular perspective that it is used to support. Pro-CL commentators drew on expert opinion and research findings to justify their support for the policy and the need for government action. Equally, framing evidence as insufficient, mixed, or negative was used to justify non-intervention.^133^ The articles we analysed selectively cited expert opinions, including medical and public health researchers, to lend authority to the perspective being communicated.^134^

 As illustrated by the conflict in perspectives between public health advocates seeking to counter rising rates of obesity and eating disorder advocates drawing attention to the potential harms of CL, there is no single public interest that can be universally appealed to when making public health policy. Policy-makers must therefore consider unintended consequences of population-level policies on marginalised publics and clearly communicate prioritisation between the needs of different publics on more than merely utilitarian grounds. It is also worth considering how business’ interests are positioned relative to those of citizens in media: portraying so-called “corporate citizens” as one legitimate voice amongst many risks concealing the radical inequality in power and resource between these entities and members of the public.^135^

 While we do not draw causal inferences between opponents’ efforts to minimise, delay, or deny the need for CL and the ultimately implemented form of the policy, it is worth noting that CL ultimately resembled the policy preferences embedded in industry-favourable framings. It excluded businesses with less than 250 employees from regulation, was not applied to alcohol, and exempted short-term specials.^136^ Corporations use media coverage to construct their anti-regulation stance as both legitimate and dominant.^137^ Our analysis demonstrates that industry-friendly language and ideology pervaded media discourse on CL, extending across policy positions. News media are powerful modes of communication that both construct and reflect public opinion.^138^ The framings they promote therefore have the potential to be politically impactful.

 CL was framed as addressing information asymmetry across the OOH environment—an ostensibly population-level policy^139^—but ultimately relied on individual consumers and businesses to make use of informational cues to generate health benefit; it is the epitome of a high-agency, population intervention.^140^ High-agency, individually-targeted interventions are ideologically appealing to libertarian and free-market perspectives because they cast interventions like CL in the quasi-voluntary language of reformulation as opposed to more “authoritarian,” mandatory legislation.^13^ Upstream individualism in policy-making highlights the persistence of lifestyle drift and failure to shift the policy paradigm away from individualism and downstream policy instruments.^2,141^ Lifestyle drift and upstream individualism are conceptually useful for understanding how much public health strategy ultimately results in lack of meaningful policy action, and media framing is one arena in which this occurs.

###  Strengths and Limitations 

 Our interpretation is strengthened by the application of established qualitative research methods. The newspapers examined have broad circulation, both in print^142^ and online,^143^ and the use of Factiva is established within media studies on public health.^45,144^ Generalisation is necessarily limited to this mode of communication and the temporal, political, and issue-specific context of our investigation.

 Quotes reported in the media, especially from organisations and their representatives, often represent prepared thoughts and intentional messages, rather than ad-hoc or conversational speech. Power relations also shape who is given a platform in these outlets. Our study therefore does not represent an exhaustive account of all framings of CL, or of the opinions or statements of the actors quoted. It does, though, offer a well-evidenced account of the principal positions adopted within the policy debate and the key interventions of the actors identified.

 Our analysis captures only what is included in 12 large, national newspapers with print editions that were indexed by Factiva and did not require significant financial or time resources to access. This study also did not include analysis of other forms of public discourse, like social media^37^ or television news coverage^145^; online-only publications; the websites of TV news channels, such as *BBC News* or *Sky News*; or local news editions, which were not indexed by Factiva. We focussed solely on English CL policy: our analysis does not extend to similar legislation under consideration at the time in Scotland due to the complex differences between devolved policy contexts. This study was also limited in its consideration of post-implementation coverage of CL, although reaching a satisfactory depth of analysis with this corpus suggested themes were well-developed.^146^

## Conclusion

 National newspaper coverage framed CL as a measure designed to address environmental information asymmetry, empower individuals to make healthier “choices,” and encourage OOH businesses to offer healthier food options. However, it also implied that individual decision-making was a matter of personal responsibility and framed people living with obesity as irresponsible and immoral. Our findings build on existing research on lifestyle drift and upstream individualism^2^ as well as connections between media framing, discursive power, and agenda setting^147^ to demonstrate that framing the policy problem as environmental does not necessarily lead to correspondingly structural solutions; instead, problems may be reconstituted through the neoliberal policy paradigm and result in correspondingly individualistic policy solutions.^2^ Framing is thus one mechanism by which lifestyle drift and upstream individualism are enacted, with the media serving as a stage for political debate. CL is a paradigmatic example of a policy that embodies the current lifestyle drift in the UK government’s obesity policy agenda. Researchers and policy advocates must be attentive to the power of framing, including through media, to shape public health policy debates. To avoid perpetuating individualism and support more effective population-level interventions, a concerted effort to reframe obesity as a structural issue and introduction of policy solutions to address social and commercial determinants of health is required.

## Ethical issues

 No ethical approval was sought for this analysis of publicly accessible news media.

## Conflicts of interest

 Authors declare that they have no conflicts of interest.

## Data availability statement

 All data is publicly accessible. A full list of news media articles used in this analysis is included in Supplementary file 2.

 For the purpose of Open Access, the authors have applied a Creative Commons Attribution (CC BY) licence to any Author Accepted Manuscript version arising.

## Endnotes


^[1]^ Several newspapers, including *The Telegraph*, *The Sun*, and *The Times*, decided to discontinue automatic publishing of sales figures in May 2020 in response to worries about a “narrative of decline.”^148^ *The Guardian* and *Observer* similarly opted out of revealing sales figures in September 2021.^142^
^[2]^ This estimated cost refers to the policy option for implementation across the OOH sector, excluding micro-businesses only.^149^
^[3]^ This estimated benefit referred to the final form of policy implementation, which applies to only large businesses with 250 or more employees. The estimated annual cost to businesses in this scenario was £0.5m.^109^

## Supplementary files


Supplementary file 1. Search Summary.

Supplementary file 2. Included Articles.

Supplementary file 3. Reflexivity Atatement.

## References

[1] Theis DRZ, White M (2021). Is obesity policy in England fit for purpose? Analysis of government strategies and policies, 1992-2020. Milbank Q.

[2] Ralston R, Godziewski C, Carters-White L (2023). The many meanings of policy instruments: exploring individual and structural determinants in obesity policy. Policy Polit.

[3] DHSC. Tackling Obesity: Empowering Adults and Children to Live Healthier Lives. GOV.UK. https://www.gov.uk/government/publications/tackling-obesity-government-strategy/tackling-obesity-empowering-adults-and-children-to-live-healthier-lives. Published July 27, 2020. Accessed June 24, 2022.

[4] van Hulst M, Yanow D (2014). From policy “frames” to “framing”: theorizing a more dynamic, political approach. Am Rev Public Adm.

[5] Entman RM (1993). Framing: toward clarification of a fractured paradigm. J Commun.

[6] Rein M, Schön D (1996). Frame-critical policy analysis and frame-reflective policy practice. Knowledge and Policy.

[7] Cullerton K, Patay D, Waller M, Adsett E, Lee A (2022). Competing public narratives in nutrition policy: insights into the ideational barriers of public support for regulatory nutrition measures. Health Res Policy Syst.

[8] Dorfman L, Wallack L, Woodruff K (2005). More than a message: framing public health advocacy to change corporate practices. Health Educ Behav.

[9] Jenkin GL, Signal L, Thomson G (2011). Framing obesity: the framing contest between industry and public health at the New Zealand inquiry into obesity. Obes Rev.

[10] Fuchs D, Lederer MM (2007). The power of business. Bus Polit.

[11] Hunter DJ, Popay J, Tannahill C, Whitehead M (2010). Getting to grips with health inequalities at last?. BMJ.

[12] Whitehead M, Popay J (2010). Swimming upstream? Taking action on the social determinants of health inequalities. Soc Sci Med.

[13] Bonell C, McKee M, Fletcher A, Haines A, Wilkinson P (2011). Nudge smudge: UK Government misrepresents “nudge. ” Lancet.

[14] Butland B, Jebb S, Kopelman P, et al. Tackling Obesities: Future Choices. London: Government Office for Science; 2007. https://assets.publishing.service.gov.uk/media/5a759da7e5274a4368298a4f/07-1184x-tackling-obesities-future-choices-report.pdf. 10.1111/j.1467-789X.2007.00344.x 17316292

[15] Sunstein CR, Thaler RH. Nudge: Improving Decisions About Health, Wealth and Happiness. Penguin Books; 2009.

[16] Hird TR, Gallagher AWA, Evans-Reeves K (2022). Understanding the long-term policy influence strategies of the tobacco industry: two contemporary case studies. Tob Control.

[17] Muggli ME, Lee K, Gan Q, Ebbert JO, Hurt RD (2008). “Efforts to Reprioritise the Agenda” in China: British American Tobacco’s efforts to influence public policy on secondhand smoke in China. PLoS Med.

[18] Hawkins B, Holden C (2013). Framing the alcohol policy debate: industry actors and the regulation of the UK beverage alcohol market. Crit Policy Stud.

[19] McCambridge J, Mialon M, Hawkins B (2018). Alcohol industry involvement in policymaking: a systematic review. Addiction.

[20] van Schalkwyk MC, Maani N, McKee M, Thomas S, Knai C, Petticrew M (2021). “When the Fun Stops, Stop”: an analysis of the provenance, framing and evidence of a ‘responsible gambling’ campaign. PLoS One.

[21] Carters-White L, Chambers S, Skivington K, Hilton S (2021). Whose rights deserve protection? Framing analysis of responses to the 2016 Committee of Advertising Practice consultation on the non-broadcast advertising of foods and soft drinks to children. Food Policy.

[22] Razavi A, Adams J, White M (2019). What arguments and from whom are most influential in shaping public health policy: thematic content analysis of responses to a public consultation on the regulation of television food advertising to children in the UK. BMJ Open.

[23] Gilmore AB, Fabbri A, Baum F (2023). Defining and conceptualising the commercial determinants of health. Lancet.

[24] Ulucanlar S, Lauber K, Fabbri A (2023). Corporate political activity: taxonomies and model of corporate influence on public policy. Int J Health Policy Manag.

[25] Harcup T, O’Neill D (2017). What is news? News values revisited (again). Journal Stud.

[26] Entman RM (1989). How the media affect what people think: an information processing approach. J Polit.

[27] McCombs ME, Shaw DL (1972). The agenda-setting function of mass media. Public Opin Q.

[28] Otten AL (1992). The influence of the mass media on health policy. Health Aff (Millwood).

[29] Weishaar H, Dorfman L, Freudenberg N (2016). Why media representations of corporations matter for public health policy: a scoping review. BMC Public Health.

[30] Rowbotham S, McKinnon M, Marks L, Hawe P (2019). Research on media framing of public policies to prevent chronic disease: a narrative synthesis. Soc Sci Med.

[31] Crockett RA, King SE, Marteau TM (2018). Nutritional labelling for healthier food or non-alcoholic drink purchasing and consumption. Cochrane Database Syst Rev.

[32] Robinson E, Marty L, Jones A, White M, Smith R, Adams J (2021). Will calorie labels for food and drink served outside the home improve public health?. BMJ.

[33] Essman M, Bishop T, Burgoine T, et al. Implementation and enforcement of mandatory calorie labelling regulations for the out-of-home sector in England: qualitative study of the experiences of business implementers and regulatory enforcers. medRxiv [Preprint]. February 20, 2024. https://www.medrxiv.org/content/10.1101/2024.02.18.24302990v1. 10.1371/journal.pone.0323452 PMC12221163 40601766

[34] Polden M, Jones A, Essman M, et al. Evaluating the effect of mandatory kilocalorie labelling on energy consumed in the out-of-home food sector: a pre vs. post-implementation observational study in England. PsyArXiv [Preprint]. February 16, 2024. https://osf.io/preprints/psyarxiv/azcqy_v1.

[35] Polden M, Jones A, Essman M (2024). Point-of-choice kilocalorie labelling practices in large, out-of-home food businesses: a preobservational versus post observational study of labelling practices following implementation of the calorie labelling (out of home sector) (England) regulations 2021. BMJ Open.

[36] DHSC. Government plans to tackle obesity in England. Department of Health and Social Care Media Centre. 2023. https://healthmedia.blog.gov.uk/2023/06/07/government-plans-to-tackle-obesity-in-england/. Accessed October 18, 2023.

[37] Polden M, Robinson E, Jones A (2023). Assessing public perception and awareness of UK mandatory calorie labeling in the out-of-home sector: using Twitter and Google trends data. Obes Sci Pract.

[38] Hope C. Online Food Delivery Companies, Restaurants and Cafes “To be Forced to Display Calorie Labels.” The Telegraph. 2018. https://www.telegraph.co.uk/politics/2018/09/14/online-food-delivery-companies-restaurants-cafes-forced-display/. Accessed May 24, 2022.

[39] Mayhew F. National Newspaper ABCs: Daily Star Sunday Sees Biggest Print Drop in First 2020 Circulation Figures. Press Gazette; 2020. https://pressgazette.co.uk/media-audience-and-business-data/media_metrics/national-newspaper-abc-daily-star-sunday-print-drop-first-2020-circulation-figures/. Accessed March 14, 2024.

[40] Braun V, Clarke V. Thematic Analysis: A Practical Guide. London: SAGE Publications; 2022.

[41] Karreman N, Essman MA, Hawkins B, Adams J, White M. Newspaper coverage of calorie labelling in the UK, 2017 – 2022: A thematic analysis. OSF [Preprint]. July 1, 2022. https://osf.io/zetgp.

[42] Birkland TA. Agenda setting in public policy. In: Fischer F, Miller GJ, Sidney MS, eds. Handbook of Public Policy Analysis: Theory, Politics, and Methods. 1st ed. Public Administration and Public Policy. Boca Raton: Taylor & Francis; 2006:63-78.

[43] Bacchi C (2016). Problematizations in health policy: questioning how “problems” are constituted in policies. Sage Open.

[44] Bacchi C. Analysing Policy: What’s the Problem Represented to Be? Frenchs Forest, NSW: Pearson Australia; 2009.

[45] Yau A, Singh-Lalli H, Forde H, Keeble M, White M, Adams J (2021). Newspaper coverage of food insecurity in UK, 2016-2019: a multi-method analysis. BMC Public Health.

[46] Braun V, Clarke V (2021). Can I use TA? Should I use TA? Should I not use TA? Comparing reflexive thematic analysis and other pattern-based qualitative analytic approaches. Couns Psychother Res.

[47] Braun V, Clarke V (2024). Supporting best practice in reflexive thematic analysis reporting in Palliative Medicine: a review of published research and introduction to the Reflexive Thematic Analysis Reporting Guidelines (RTARG). Palliat Med.

[48] DHSC. Childhood Obesity: A Plan for Action, Chapter 2. UK: HM Government; 2018. https://assets.publishing.service.gov.uk/media/5b30a40de5274a55c78cef32/childhood-obesity-a-plan-for-action-chapter-2.pdf.

[49] Davies J. Revealed, the Most Calorific Meals at UK’s Biggest Chains: Fried Breakfasts at Toby Carvery Contain Up to 2,400 Calories (And Even A “Cheeky Nando’s” Can Eat Up Almost All of Your Daily Limit). Daily Mail; 2022. https://www.dailymail.co.uk/health/article-10687089/The-calorific-meals-UKs-biggest-restaurant-chains.html. Accessed May 24, 2022.

[50] Duffield C. Calorie Counts Set to Appear on Menus as Part of Government Drive to Tackle Obesity. The Independent; 2022. https://www.independent.co.uk/life-style/food-and-drink/calorie-counts-menus-obsesity-b2051305.html. Accessed May 24, 2022.

[51] Quinn S. “The Bottom Line is That You Don’t Need to Count Calories.” The Daily Telegraph. 2020:21.

[52] Waugh D. Calorie counts on menus will not make any of us thinner. i. 2018:22.

[53] Elgot J. Web Ads for Junk Food Could be Banned Under UK Government Plans. The Guardian. 2020:15.

[54] Barr S. What is Obesity and How is it Measured? The Independent; 2020. https://www.independent.co.uk/life-style/health-and-families/obesity-definition-bmi-government-bogof-junk-food-health-a9639776.html. Accessed May 24, 2022.

[55] Clarke J. Calorie Counts to Appear on Menus as Part of Government Drive to Tackle Obesity. The Standard; 2022. https://www.standard.co.uk/news/uk/government-businesses-england-obesity-diners-b992589.html. Accessed May 24, 2022.

[56] Gallagher S. ‘I Would Think - Best Not Eat Then’: Why Putting Exercise Labels on Food Could Impact People with Eating Disorders. The Independent; 2020. https://www.independent.co.uk/life-style/food-exercise-labels-calories-obesity-eating-disorders-a9241906.html. Accessed May 24, 2022.

[57] Grant K. How fast food took over our high streets. i. 2018:24,25.

[58] Donnelly L. Most Restaurant Diners Want Calorie Counts on the Menu. The Telegraph; 2018. https://www.telegraph.co.uk/global-health/climate-and-people/restaurant-diners-want-calorie-counts-menu/. Accessed May 24, 2022.

[59] Ng K. Would More Labelling Really Help Us Eat Less Sugar? The Independent; 2021. https://www.independent.co.uk/life-style/food-and-drink/sugar-consumption-labels-obesity-health-b1904772.html. Accessed May 24, 2022.

[60] Watson S. Want to Lose Weight? Turn the Heating Off and Book a Photographer. The Daily Telegraph. 2018:24.

[61] Wooller S. Bojo’s Battle of the Bulge. The Sun. 2020:2.

[62] Boyd C. Revealed: The Pancakes, Waffles and Crepes That Contain Three Times More Calories Than a Big Mac and as Much Sugar as Two Cans of Coke. Daily Mail; 2019. https://www.dailymail.co.uk/health/article-7451851/The-pancakes-waffles-crepes-contain-THREE-TIMES-calories-Big-Mac.html. Accessed May 24, 2022.

[63] Pickles K. Coffee Shops in the Dock: Health Chiefs Warns High Street Chains’ “Little Nudges” to Buy Sweet Treats Are Fuelling Obesity Crisis. Daily Mail; 2018. https://www.dailymail.co.uk/health/article-6170229/Health-chiefs-warns-high-streets-little-nudges-buy-sweet-treats-fuelling-obesity-crisis.html. Accessed May 24, 2022.

[64] Boyd C. Forcing Restaurants to Put Nutritional Information on Their Menus Shames Them into Providing Healthier Food, Cambridge Study Finds. Daily Mail; 2019. https://www.dailymail.co.uk/health/article-7579699/Nutritional-information-menus-shames-firms-providing-healthier-food-Cambridge-study-finds.html. Accessed May 24, 2022.

[65] Borland S. Nation Gorging on Fast Food: Record Surge in Takeaways on Almost Every High Street is Feeding Britain’s Crippling Obesity Crisis. Daily Mail; 2018. https://www.dailymail.co.uk/news/article-6309241/Record-surge-takeaways-high-street-feeding-Britains-crippling-obesity-crisis.html. Accessed May 24, 2022.

[66] Donnelly L. Putting Calories on the Menu Could Help to Cut Obesity. The Daily Telegraph. 2018:6.

[67] Allen V, Spencer B. Women Eat 75 Fewer Calories if Nutritional Information is Printed on Menus, Study Finds. Daily Mail; 2017. https://www.dailymail.co.uk/health/article-5059079/Calorie-counts-don-t-work-men-study-finds.html. Accessed May 24, 2022.

[68] Borland S. Takeaway customers ordering with Deliveroo will see meal calories in a drive to encourage healthier eating. Daily Mail; 2019. https://www.dailymail.co.uk/health/article-6609097/Takeaway-firm-meal-calories-drive-encourage-healthier-eating.html. Accessed May 24, 2022.

[69] Jackson A. Obesity and Health is Not One-Size-Fits-All – And Solutions Like Calorie Shaming Do Not Work! The Independent; 2021. https://www.independent.co.uk/life-style/health-and-families/obesity-nhs-digital-england-regulation-b1849392.html. Accessed May 24, 2022.

[70] The Telegraph. Letters: Archbishop of Canterbury Wants Higher Taxes, But Not for His Church. The Telegraph; 2018. https://www.telegraph.co.uk/opinion/2018/09/06/lettersarchbishop-canterbury-wants-higher-taxes-not-church/. Accessed May 24, 2022.

[71] Wilson A. Why Boris Johnson’s Obesity Campaign Could Cause Further Suffering for Those with Eating Disorders. The Independent; 2020. https://www.independent.co.uk/voices/boris-johnson-better-health-obesity-campaign-coronavirus-menus-calories-b447407.html. Accessed May 24, 2022.

[72] Feehan K. Hospitality Bosses Say Calorie Counts on Menus “Come at the Worst Possible Time” for Pubs and Restaurants Struggling to Survive After Pandemic - As Diners Say They Feel Too Guilty to Eat Out Just a Week After Strict New Rules. Daily Mail; 2022. https://www.dailymail.co.uk/news/article-10715063/Pub-restaurant-bosses-say-calorie-counts-menus-come-worst-possible-time-pandemic.html. Accessed May 24, 2022.

[73] Simmons E. We Don’t Need Ocado Nagging Us to Cut the Calories in Our Weekly Shop. Daily Mail; 2019. https://www.dailymail.co.uk/health/article-7390539/EVE-SIMMONS-dont-need-Ocado-nagging-cut-calories-weekly-shop.html. Accessed May 24, 2022.

[74] The Sun. The Sun Says: Weight a Minute. The Sun. 2017:10.

[75] Finney C. Putting Calories on Menus Won’t Solve Obesity, But it Will Harm Those of Us With Eating Disorders. The Guardian; 2022. https://www.theguardian.com/commentisfree/2022/apr/01/calories-menus-obesity-eating-disorders. Accessed May 24, 2022.

[76] Gallagher S. Doesn’t Weigh Up. The Independent. 2020:34.

[77] Abdul G. ‘I’m Responsible for What I Put in My Body’: Consumers Respond to Calorie Labelling Rollout. The Guardian. 2022:27.

[78] Dollimore L. Restaurant Chains Print Calorie Counts on Menus Today: Wahaca and Wagamama Bosses and No10 Food Tsar Warn it Won’t Tackle Obesity Because it Doesn’t Show Difference Between Good and Bad Calories - As Charities Fear a Rise in Eating Disorders. Daily Mail; 2022. https://www.dailymail.co.uk/news/article-10691235/Restaurant-chains-print-calorie-counts-menus-TODAY-industry-leader-say-WONT-tackle-obesity.html. Accessed May 24, 2022.

[79] The Daily Mail. 3 in 4 Want All Restaurants to Show Calories. Scotland: The Daily Mail. 2018:43.

[80] Stern C. Trying to Stay Healthy? Don’t Get Duped by the Menu! The Seven Sneaky Things to Avoid When Dining Out at a Restaurant, from Anything “Crispy” or “Creamy” to Misleading Salads. Daily Mail; 2018. https://www.dailymail.co.uk/femail/article-5935691/7-sneaky-things-things-avoid-dining-diet.html. Accessed May 24, 2022.

[81] Cowburn A. Government Launches New Measures to Tackle Obesity. The Independent. 2020:8.

[82] Tapsfield J. Boris Pushes Ahead with “Nanny State” Total Ban on Online Junk Food Ads After His Own Covid Battle - Amid Fears Avocados, Salmon, Marmite and Houmos Could Fall Foul of New Rules. Daily Mail; 2021. https://www.dailymail.co.uk/news/article-9565817/Ban-online-junk-food-ads-ahead.html. Accessed May 24, 2022.

[83] Webber E. Conservatives Struggle with Their Body Image. The Times; 2020. https://www.thetimes.co.uk/article/conservatives-struggle-with-their-body-image-ndvjzn79l. Accessed May 24, 2022.

[84] Matthews S. Could Adverts for Butter, Cheese and Tomato Ketchup be Banned Before 9pm? Pre-Watershed Crackdown on “HFSS” Food Commercials Will Hit Many Foods “No Reasonable Person Would Consider Unhealthy.” Daily Mail; 2020. https://www.dailymail.co.uk/news/article-8564823/Say-goodbye-adverts-butter-cheese-tomato-ketchup.html. Accessed May 24, 2022.

[85] Donnelly L, Hymas C. Minister Triggers Backlash Against Counting Calories. The Daily Telegraph. 2018:6.

[86] Coleman C. Revealed: Shocking Truth About Those Calorie-Counted Supermarket Meals. Daily Mail. 2022:42,43.

[87] Geissler H. Cafes Urged to Show Unhealthy Food Alerts. The Daily Express. 2019:10.

[88] Harvey G. Junk Food Isn’t the Only Enemy in the Fight Against Obesity – It’s the Products Which Pretend to be Healthy. The Independent; 2020. https://www.independent.co.uk/voices/obesity-crisis-calories-menus-food-industry-labelling-boris-johnson-a9641611.html. Accessed May 24, 2022.

[89] Smith G. Calorie counts turn dining out into a maths problem. i. 2019:22.

[90] Wallace G. Calorie Info on Menus...Help to Lose Weight or Too Much Nanny State? The Sun. 2021:26.

[91] Pearson-Jones B. Michelin-Starred Chefs Lead Backlash to Calorie Counts on Menus as They Are Introduced in Government Scheme, Saying it Encourages Eating Disorders and Will “Lead to Boring, Tick-Box Cooking.” Daily Mail; 2022. https://www.dailymail.co.uk/femail/food/article-10669857/Chefs-lead-backlash-against-calorie-counts-menus.html. Accessed May 24, 2022.

[92] Wooller S. Will Putting Meals’ Calorie Counts on Menu Make You Eat More Healthily? Half of Diners Are Likely to Order More Nutritious Dishes When Restaurants Are Forced to Introduce the Measure This Week, Study Suggests. Daily Mail; 2022. https://www.dailymail.co.uk/news/article-10682429/Will-putting-meals-calorie-counts-menu-make-eat-healthily.html. Accessed May 24, 2022.

[93] DHSC. New Calorie Labelling Rules Come into Force to Improve Nation’s Health. GOV.UK website. https://www.gov.uk/government/news/new-calorie-labelling-rules-come-into-force-to-improve-nations-health. Published April 6, 2022. Accessed June 24, 2022.

[94] Theis DR, Adams J (2019). Differences in energy and nutritional content of menu items served by popular UK chain restaurants with versus without voluntary menu labelling: a cross-sectional study. PLoS One.

[95] Meade A. Calorie counts threaten my eating disorder recovery. i. 2021:21.

[96] Boycott-Owen M. Labelling Food with Exercise to Burn it Off “Can Curb Obesity.” The Daily Telegraph. 2019:10.

[97] Henderson E. The Cost of Counting. The Independent. 2022:3.

[98] Horton H. Calories on Restaurant Menus Will Fuel Young Peoples’ Eating Disorders, Charities and MPs Warn Government. The Telegraph; 2018. https://www.telegraph.co.uk/news/2018/12/08/calories-restaurant-menus-will-fuel-young-peoples-eating-disorders/. Accessed May 24, 2022.

[99] The Daily Telegraph. Fat Chance. The Daily Telegraph. 2018:21.

[100] Petter O. Calorie Counts on Restaurant Menus Reduce How Much People Eat by 12%, Study Finds. The Independent; 2018. https://www.independent.co.uk/life-style/food-and-drink/calorie-counts-restaurant-menus-reduce-people-eat-study-a8230981.html. Accessed May 24, 2022.

[101] Shah F. Experts Warn Move to Calorie-Labelled Menus is ‘Problematic’. The Independent; 2022. https://www.independent.co.uk/life-style/food-and-drink/calorie-counting-on-menu-england-experts-b2052804.html. Accessed May 24, 2022.

[102] Boyd C. Fury over Matt Hancock’s “un-British” Plan to Slap Calorie Counts on Beer, Wine and Spirits (But do You Really Want to Know How Fattening Your Favourite Tipple Is?). Daily Mail; 2021. https://www.dailymail.co.uk/news/article-9470273/Fury-No10s-British-plan-slap-calorie-counts-beer-wine-spirits.html. Accessed May 24, 2022.

[103] Dunn TN. Scoff the Menu. The Sun. 2019:16.

[104] Portman Group. About us. https://www.portmangroup.org.uk/. Accessed January 22, 2024.

[105] Grant J. Pubs Will Not be Forced to List Calories on Booze! Government Drops Obesity-Busting Plan to Put Health Warnings on Alcohol Due to Industry “On its Knees.” Daily Mail; 2021. https://www.dailymail.co.uk/news/article-9567189/Pubs-NOT-forced-list-calories-booze-Government-drops-obesity-busting-plan.html. Accessed May 24, 2022.

[106] Hughes L. Calorie Labelling on Menus Too ‘Burdensome’, Warns Treasury. Financial Times; 2018. https://www.ft.com/content/d1c906ee-b11a-11e8-99ca-68cf89602132. Accessed May 24, 2022.

[107] Chalmers V, Pyman T. Obese People Are More Than Three Times as Likely to Die of COVID-19 and Seven Times More Likely to Need a Ventilator, PHE Reveals as Boris Launches National Diet Crusade. Daily Mail; 2020. https://www.dailymail.co.uk/news/article-8557629/Overweight-people-THREE-TIMES-likely-die-Covid-19.html. Accessed May 24, 2022.

[108] Smyth C. Ban on Junk Food Adverts Likely Within Two Years. The Times.2020:11.

[109] DHSC. Mandating Calorie Labelling of Food and Drink in Out-of-Home Settings. Department of Health and Social Care; 2020. https://assets.publishing.service.gov.uk/government/uploads/system/uploads/attachment_data/file/992872/calorie-labelling-impact-assessment.pdf.

[110] Swinford S. Plan to Force All Restaurants, Cafes and Takeaways to Display Calorie Counts on Menus Sparks Cabinet Row. The Telegraph; 2018. https://www.telegraph.co.uk/politics/2018/09/04/plans-force-restaurants-cafes-takeaways-display-calorie-counts/. Accessed May 24, 2022.

[111] Uttley H. Struggling Restaurants Face Fresh Hammer Blow from Menu Calorie Rule. The Telegraph; 2020. https://www.telegraph.co.uk/business/2020/07/28/struggling-restaurants-face-fresh-hammer-blow-menu-calorie-rule/. Accessed May 24, 2022.

[112] Wright O. Treasury Hits Back Over Healthier Menus. The Times. 2018:4.

[113] Ellson A. Brewers Furious at “Nanny State” Plan to Put Calorie Count on Beer. The Times. 2021:8.

[114] Swan TM. Pub Landlords: Beer Pump Calorie Counts Are “A Creeping Nanny State on Steroids.” The Telegraph; 2021. https://www.telegraph.co.uk/food-and-drink/news/creeping-nanny-state-steroids-pub-landlords-rail-against-calorie/. Accessed May 24, 2022.

[115] Donnelly L. Calorie Labels Will be Placed on Millions of Takeaway Menus. The Telegraph; 2019. https://www.telegraph.co.uk/news/2019/01/18/calorie-labels-will-placed-millions-takeaway-menus/. Accessed May 24, 2022.

[116] Moore C. The Curse of Childhood Obesity Begins at Home. The Telegraph; 2018. https://www.telegraph.co.uk/news/2018/06/24/curse-childhood-obesity-begins-home/. Accessed May 24, 2022.

[117] Wheeler C. Ministers Mull Ban on Sale of Energy Drinks to Children. The Sunday Times. 2018:2.

[118] Moodie C. Calories on Menu Will Just Drive Us All Nandoolally. The Sun. 2022:15.

[119] Money-Coutts S. We’ve All Gone Barking Mad Over Our Dogs (There, I’ve Said it). The Telegraph; 2022. https://www.telegraph.co.uk/columnists/2022/05/15/gone-barking-mad-dogs-said/. Accessed May 24, 2022.

[120] Odell M, Evans H. Calories on Menus? Date Night is Ruined! The Times; 2022. https://www.thetimes.co.uk/article/calories-on-menus-date-night-is-ruined-7vsck0zpg. Accessed May 24, 2022.

[121] Simmons E. Why Putting Calories on Restaurant Menus Fills Me With Fear. Daily Mail; 2020. https://www.dailymail.co.uk/health/article-8607267/EVE-SIMMONS-putting-calories-restaurant-menus-fills-fear.html. Accessed May 24, 2022.

[122] Deacon M. Jamie Oliver Wants to Ban Cheap Junk Food Deals During a Cost of Living Crisis? Frankly, He Can BOGOF. The Daily Telegraph. 2022:6,7.

[123] Wilcock D, Mailonline WCF. “Sin Tax” on Sugary Fizzy Drinks Could be Extended to Chocolates with Adverts for Sugary Treats Banned and Health Warnings Slapped on Alcohol Bottles in Anti-Obesity Plans Being Considered by Boris Johnson. Daily Mail; 2020. https://www.dailymail.co.uk/news/article-8565253/Sin-tax-sugary-fizzy-drinks-extended-chocolates-sweets.html. Accessed May 24, 2022.

[124] LeBesco K (2011). Neoliberalism, public health, and the moral perils of fatness. Crit Public Health.

[125] Hawkins B, van Schalkwyk MC. Discourses of Balance in the Corporate Political Strategies of Health Harming Industries: A Comparative Analysis of the Gambling and Opioid Industries. In: T09P06 Commercial Political Actors in Comparative Perspective. Toronto, Canada: International Public Policy Association; 2023. https://www.ippapublicpolicy.org//file/paper/648c95b9e09c9.pdf.

[126] Room R (2011). Addiction and personal responsibility as solutions to the contradictions of neoliberal consumerism. Crit Public Health.

[127] Hay P, Mitchison D (2019). Eating disorders and obesity: the challenge for our times. Nutrients.

[128] Herndon AM (2005). Collateral damage from friendly fire?: Race, nation, class and the “war against obesity. ” Soc Semiot.

[129] O’Hara L, Gregg J (2006). The war on obesity: a social determinant of health. Health Promot J Austr.

[130] Parkhurst J, Ettelt S, Hawkins B. Evidence Use in Health Policy Making: An International Public Policy Perspective. Cham: Springer International Publishing; 2018. doi: 10.1007/978-3-319-93467-9.

[131] Hawkins B, Parkhurst J (2016). The ‘good governance’ of evidence in health policy. Evid Policy.

[132] Petticrew M, Katikireddi SV, Knai C (2017). ‘Nothing can be done until everything is done’: the use of complexity arguments by food, beverage, alcohol and gambling industries. J Epidemiol Community Health.

[133] Ulucanlar S, Fooks GJ, Gilmore AB (2016). The policy dystopia model: an interpretive analysis of tobacco industry political activity. PLoS Med.

[134] Zelizer B (1989). ‘Saying’ as collective practice: quoting and differential address in the news. Text - Interdisciplinary Journal for the Study of Discourse.

[135] Nyberg D, Murray J (2020). Corporate politics in the public sphere: corporate citizenspeak in a mass media policy contest. Bus Soc.

[136] HM Government. The Calorie Labelling (Out of Home Sector) (England) Regulations 2021. No. 909. King’s Printer of Acts of Parliament; 2021. https://www.legislation.gov.uk/uksi/2021/909/contents/made. Accessed January 28, 2024.

[137] Murray J, Nyberg D (2021). Industry vs government: leveraging media coverage in corporate political activity. Organ Stud.

[138] Wlezien C, Soroka S (2024). Media reflect! Policy, the public, and the news. Am Polit Sci Rev.

[139] Rose G. Rose’s strategy of preventive medicine. In: The Population Strategy of Prevention. Oxford University Press. 2008:129-140.

[140] Garrott K, Ogilvie D, Panter J (2024). Development and application of the Demands for Population Health Interventions (Depth) framework for categorising the agentic demands of population health interventions. BMC Glob Public Health.

[141] Schmidt VA (2016). The roots of neo-liberal resilience: explaining continuity and change in background ideas in Europe’s political economy. Br J Polit Int Relat.

[142] Tobitt C, Majid A. National Press ABCs: FT Sees Biggest Month-on-Month Print Fall in February. Press Gazette; 2024. https://pressgazette.co.uk/media-audience-and-business-data/media_metrics/most-popular-newspapers-uk-abc-monthly-circulation-figures-2/. Accessed March 15, 2024.

[143] Majid A. Top 50 UK News Websites: Money Saving Expert and Telegraph See Double-Digit Growth in January. Press Gazette; 2024. https://pressgazette.co.uk/media-audience-and-business-data/media_metrics/most-popular-websites-news-uk-monthly-2/. Accessed March 15, 2024.

[144] Penney TL, Jones CP, Pell D (2023). Reactions of industry and associated organisations to the announcement of the UK Soft Drinks Industry Levy: longitudinal thematic analysis of UK media articles, 2016-18. BMC Public Health.

[145] O’Sullivan T, Daniel E, Harris F (2023). Media and the staging of policy controversy: obesity and the UK sugar tax. Crit Policy Stud.

[146] Malterud K, Siersma VD, Guassora AD (2016). Sample size in qualitative interview studies: guided by information power. Qual Health Res.

[147] Roslyng MM, Dindler C (2023). Media power and politics in framing and discourse theory. Commun Theory.

[148] BBC. ABC Figures: Newspapers Will No Longer Have to Publish Sales. BBC News; 2020. https://www.bbc.com/news/entertainment-arts-52754762. Accessed March 14, 2024.

[149] DHSC. Mandating Energy Labelling of Food and Drink in Out-of-Home Settings. HM Government; 2018. https://assets.publishing.service.gov.uk/government/uploads/system/uploads/attachment_data/file/751532/impact-assessment-for-consultation-on-calorie-labelling-outside-of-the-home.pdf.

