# Supplementary material for: Obesity and Lifestyle Drift: Framing Analysis of Calorie Menu Labelling in England in News Media
Source: Int J Health Policy Manag. 2025 Apr 28;14:8649. doi: 10.34172/ijhpm.8649 (PMC12257196; doi:10.34172/ijhpm.8649)
Supplement: Supplementary file 1 — Search Summary. [file ijhpm-14-8649-s001.pdf]

Article title: Obesity and Lifestyle Drift: Framing Analysis of Calorie Menu Labelling in England in News Media

Journal name: International Journal of Health Policy and Management (IJHPM)

Authors' information: Nancy Karreman*, Michael Essman, Benjamin Hawkins, Jean Adams, Martin White

MRC Epidemiology Unit, University of Cambridge, Cambridge, UK

***Correspondence to:** Nancy Karreman; Email: nancy.karreman@mrc-epid.cam.ac.uk

Citation: Karreman N, Essman M, Hawkins B, Adams J, White M. Obesity and lifestyle drift: framing analysis of calorie menu labelling in England in news media. Int J Health Policy Manag. 2025;14:8649. doi:10.34172/ijhpm.8649

Supplementary file 1. Search Summary

Supplemental Table S1: Summary of search generated from Factiva on 24 May 2022.

**Search Summary**	
**Text**	calori* and menu* and label*
**Date**	In the last 5 years
**Source**	The Independent - All sources Or The Guardian (U.K.) Or The Observer (U.K.) Or The Daily Mirror (U.K.) Or The Sunday Mirror (U.K.) Or The Sun (U.K.) Or Sunday Sun Or The Times (U.K.) - All sources Or The Daily Express (U.K.) Or Sunday Express (U.K.) Or Financial Times (Available through Third Party Subscription Services) - All sources Or Daily Star (U.K.) Or Daily Star Sunday (U.K.) Or Sunday People (U.K.) Or Morning Star (U.K.) Or Daily Mail - All sources Or The Telegraph (U.K.) - All sources
**Author**	All Authors
**Company**	All Companies
**Subject**	All Subjects
**Industry**	All Industries
**Region**	United Kingdom; NOT Scotland
**Language**	English
**Results Found**	650
**Timestamp**	24 May 2022 13:45
