# Supplementary material for: Obesity and Lifestyle Drift: Framing Analysis of Calorie Menu Labelling in England in News Media
Source: Int J Health Policy Manag. 2025 Apr 28;14:8649. doi: 10.34172/ijhpm.8649 (PMC12257196; doi:10.34172/ijhpm.8649)
Supplement: Supplementary file 2 — Included Articles. [file ijhpm-14-8649-s002.pdf]

Article title: Obesity and Lifestyle Drift: Framing Analysis of Calorie Menu Labelling in England in News Media

Journal name: International Journal of Health Policy and Management (IJHPM)

Authors' information: Nancy Karreman*, Michael Essman, Benjamin Hawkins, Jean Adams, Martin White

MRC Epidemiology Unit, University of Cambridge, Cambridge, UK

***Correspondence to:** Nancy Karreman; Email: nancy.karreman@mrc-epid.cam.ac.uk

Citation: Karreman N, Essman M, Hawkins B, Adams J, White M. Obesity and lifestyle drift: framing analysis of calorie menu labelling in England in news media. Int J Health Policy Manag. 2025;14:8649. doi:10.34172/ijhpm.8649

Supplementary file 2. Included Articles

Supplemental Table S2: Articles included in analysis.

Author	Title	Publication	Date
Abdul, G.	'I'm responsible for what I put in my body': Consumers respond to calorie labelling rollout	The Guardian	07/04/2022
Abraham, E.	Adding calories to menus will harm those with eating disorders, charity warns	Independent Online	25/05/2021
Allen, V.; Spencer, B.	Women eat 75 FEWER calories if nutritional information is printed on menus, study finds	Mail Online	07/11/2017
Barker, A.; Neville, S.; Evans, J.; Parker, G.	Johnson to impose curbs on ads for junk food amid anti-obesity drive	Financial Times	24/07/2020
Barr, S.	What is obesity and how is it measured?	Independent Online	27/07/2020
Barrie, J.	Calls to delay calorie labelling by six months	i	23/06/2021
Borland, S.	Nation gorging on fast food: Record surge in takeaways on almost every high street is feeding Britain's crippling obesity crisis	Mail Online	23/10/2018
Borland, S.	Takeaway customers ordering with Deliveroo will see meal calories in a drive to encourage healthier eating	Mail Online	19/01/2019
Boycott-Owen, M.	Labelling food with exercise required to burn it off 'can curb obesity'	The Telegraph Online	11/12/2019

Boyd, C.	Government accused of a 'cop out' after leak shows plan to place calorie labels in all restaurants, cafes and takeaways will only apply to 520 businesses	Mail Online	06/06/2019
Boyd, C.	Revealed: The pancakes, waffles and crepes that contain THREE TIMES more calories than a Big Mac and as much sugar as two cans of Coke	Mail Online	12/09/2019
Boyd, C.	Global obesity epidemic laid bare: The average life expectancy of Americans is almost FOUR YEARS shorter, and for Britons it's nearly three years, thanks to soaring deaths	Mail Online	10/10/2019
Boyd, C.	Forcing restaurants to put nutritional information on their menus shames them into providing healthier food, Cambridge study finds	Mail Online	16/10/2019
Boyd, C.	Labelling chocolate and crisps with the amount of exercise needed to burn them off shames people into eating 200 less calories a day (so how long would you need to run for to burn off your favourite treat?)	Mail Online	11/12/2019
Boyd, C.	Fury over Matt Hancock's 'un-British' plan to slap calorie counts on beer, wine and spirits (but do you really want to know how fattening your favourite tipple is?)	Mail Online	14/04/2021
Boyd, C.	Banning alcohol from being sold in supermarkets and making it available only in off-licences could clamp down on Britain's booze crisis, experts say	Mail Online	19/04/2021
Boyd, C.	Boris Johnson ditches nanny state ban on BOGOF junk food deals to soften blow of cost of living crisis	Mail Online	13/05/2022
Buaya, A.	Takeaways, small restaurants and greasy spoon cafes will be spared having to list calorie counts on their menus	Mail Online	10/05/2019
Burgess, K.	Why calories on the menu makes for healthier meals	The Times	17/10/2019
Burgess, K.	Effect of calories on menus fades away	The Times	31/10/2019
Burgess, K.	Junk food labels to show how much that moment on the lips really costs	The Times	11/12/2019
Chalmers, V.	Use traffic light labels and charge more for them in restaurants: Health experts create an eight-point blueprint for how governments can cut down fizzy drinks to fight childhood obesity	Mail Online	12/06/2019

Chalmers, V.; Pyman, T.	Obese people are more than THREE TIMES as likely to die of Covid-19 and seven times more likely to need a ventilator, PHE reveals as Boris launches national diet crusade	Mail Online	25/07/2020
Clarke, J.	Calorie counts to appear on menus as part of Government drive to tackle obesity	Independent Online	05/04/2022
Clarke, J.	Calorie count on menus in obesity fight	The Daily Mirror	06/04/2022
Cole, H.	Calories label bid for your pub pint	The Sun	14/04/2021
Cole, H.	Brew serious?	The Sun	15/04/2021
Coleman, C.	Revealed: Shocking truth about those calorie-counted supermarket meals	Daily Mail	11/04/2022
Cowburn, A.	Restaurants to include calorie counts as Boris Johnson cracks down on obesity crisis	Independent Online	27/07/2020
Cowburn, A.	Government launches new measures to tackle obesity	The Independent	27/07/2020
Cowburn, A.	Pints and drinks won’t be included in plans to force hospitality to introduce calorie labels, No 10 suggests	Independent Online	11/05/2021
Cracknell, J.	Winning is being healthy and able to enjoy your life	Sunday Express	02/08/2020
Davies, J.	Revealed, the most calorific meals at UK's biggest chains: Fried breakfasts at Toby Carvery contain up to 2,400 CALORIES (and even a 'cheeky Nando's' can eat up almost all of your daily limit)	Mail Online	05/04/2022
Davis, N.	Exercise advice on food labels could help to tackle the obesity crisis	The Guardian	10/12/2019
Deacon, M.	Jamie Oliver wants to ban deals on junk food. Frankly, he can BOGOF	The Daily Telegraph	19/05/2022
Dilworth, M.; Sculthorpe, T.	British restaurants could be forced to display calorie counts on their menus despite concerns it could lead to price rises and job cuts	Mail Online	05/09/2018
Dollimore, L.	Restaurant chains print calorie counts on menus TODAY: Wahaca and Wagamama bosses and No10 food tsar warn it WON'T tackle obesity because it doesn't show difference between good and bad calories - as charities fear a rise in eating disorders	Mail Online	06/04/2022
Donnelly, L.	Putting calories on the menu could help to cut obesity	The Daily Telegraph	27/02/2018
Donnelly, L.	Calories on the menu make diners think twice - cutting intake by 12 %	The Telegraph Online	27/02/2018

Donnelly, L.	Most restaurant diners want calorie counts on the menu	The Telegraph Online	21/05/2018
Donnelly, L.	Restaurants must reveal calorie counts to fight child obesity	The Sunday Telegraph	24/06/2018
Donnelly, L.	Calorie labels will be placed on millions of takeaway menus	The Telegraph Online	18/01/2019
Donnelly, L.; Hymas, C.	Minister triggers backlash against counting calories	The Daily Telegraph	27/12/2018
Donnelly, L.; Meadows, S.; Yorke, H.	Exclusive: Calories on pints and bottles of wine as drive against obesity targets alcohol	The Telegraph Online	24/07/2020
Donnelly, L.; Rayner, G.	Lose 5lbs and save NHS £100m, Matt Hancock says after UK's coronavirus 'wake-up call' on obesity	The Telegraph Online	27/07/2020
Duchess, S.	Being called Duchess of Pork hurt but I won my weight battle, and this country can too	The Sun	11/11/2020
Duffield, C.	Calorie counts set to appear on menus as part of government drive to tackle obesity	Independent Online	05/04/2022
Dunn, Tom N.	Scoff the menu	The Sun	18/01/2019
Elgot, J.	Web ads for junk food could be banned under UK government plans	The Guardian	27/07/2020
Elliott, F.	Calories to be displayed on all menus	The Times	14/09/2018
Ellson, A.	Brewers furious at 'nanny state' plan to put calorie count on beer	The Times	15/04/2021
Ellson, A.; Zeffman, H.	Child obesity action plan 'is too feeble' [CORRECTED]	The Times	25/06/2018
Feehan, K.	Hospitality bosses say calorie counts on menus 'come at the worst possible time' for pubs and restaurants struggling to survive after pandemic - as diners say they feel too guilty to eat out just a week after strict new rules	Mail Online	13/04/2022
Finney, C.	Putting calories on menus won’t solve obesity, but it will harm those of us with eating disorders	The Guardian	06/04/2022
Forsyth, L.	Restaurants, kebab shops and cafes will now be forced to reveal calorie counts under new government plans to halve childhood obesity in 12 years	Mail Online	24/06/2018
Gallagher, S.	‘I would think -best not eat then’: Why putting exercise labels on food could impact people with eating disorders	Independent Online	27/07/2020

Gallagher, S.	Doesn't weigh up	The Independent	04/08/2020
Gant, J.	Pubs will NOT be forced to list calories on booze! Government drops obesity-busting plan to put health warnings on alcohol due to industry 'on its knees'	Mail Online	12/05/2021
Geissler, H.	Cafes urged to show unhealthy food alerts	The Daily Express	22/01/2019
Grant, K.	How fast food took over our high streets	i	03/11/2018
Grant, K.	Calorie labelling legislation 'a risk to businesses'	i	11/02/2020
Groves, J.	Calorie labels on alcohol and your night out	Daily Mail	25/07/2020
Hampson, L.	Why have calorie labels been introduced on menus?	Independent Online	06/04/2022
Harvey, G.	Junk food isn't the only enemy in the fight against obesity – it's the products which pretend to be healthy	Independent Online	28/07/2020
Henderson, E.	The cost of counting	The Independent	09/04/2022
Hope, C.	Online food delivery companies, restaurants and cafes 'to be forced to display calorie labels'	The Telegraph Online	14/09/2018
Horton, H.	Calories on restaurant menus will fuel young peoples' eating disorders, charities and MPs warn government	The Telegraph Online	08/12/2018
Horton, I.	'Doomed to fail', plans to tackle UK's obesity crisis	Scottish Daily Mail	28/12/2021
Hughes, L.	Calorie labelling on menus too 'burdensome', warns Treasury	Financial Times (FT.Com)	05/09/2018
Hughes, L.; Neville, S.	Online junk food ads face total UK ban in drive to tackle obesity	Financial Times (FT.Com)	27/07/2020
i	Controversy freakshake ban	i	17/11/2018
Jackson, A.	Obesity and health is not one-size-fits-all – and solutions like calorie shaming do NOT work!	Independent Online	18/05/2021
Kingsley, T.	Calls for meal deals to be banned from Tesco, M&S, Boots, Sainsbury's and Asda	Independent Online	14/05/2022
Lawton, J.	Fats food diners	Daily Star	28/05/2018
Lay, K.	Calorie counts really take the biscuit	The Times	27/02/2018

Lewis, L.	Councils could refuse to licence bars and clubs in a bid to reduce hospital admissions as they push to have 'public health' added to their remit to 'prepare for a future pandemic'	Mail Online	07/08/2021
Linning, S.	Revealed: How a quarter of high street restaurant chains provide NO details of calorie content to customers - and only WETHERSPOONS lists the information on its menu	Mail Online	03/05/2018
Lister, S.	Boris blueprint for victory in the war on obesity	The Daily Express	27/07/2020
Logan, J.	Menus with calorie details 'are lower in fat'	i	17/10/2019
Lytton, C.	One less Snickers ad won't solve the obesity crisis	The Daily Telegraph	25/06/2021
Maidment, J.	Lose weight, save the NHS: Boris Johnson reveals his fight to diet as government launches anti-obesity drive	Mail Online	27/07/2020
Matthews, S.	Could adverts for butter, cheese and tomato ketchup be banned before 9pm? Pre-watershed crackdown on 'HFSS' food commercials will hit many foods 'no reasonable person would consider unhealthy'	Mail Online	27/07/2020
McDermott, N.	Calories on menu cut food sale rates	The Sun	31/10/2019
McDermott, N.	Weight walkers	The Sun	11/12/2019
McDermott, N.	You're round	The Sun	15/07/2020
Meade, Aimee	Calorie counts threaten my eating disorder recovery	i	14/05/2021
Meadows, S.	Lab grown meat should be funded by the Government to help beat obesity, think tank says	The Telegraph Online	10/08/2020
Middleton, J.	Look away Boris! Postcode checker shows which pubs and restaurants are offering up to £10 off meals in August under Rishi Sunak's new scheme - and it doesn't look like it will help much with the government's anti-obesity drive!	Mail Online	28/07/2020
Money-Coutts, S.	We've all gone barking mad over our dogs (There, I've said it)	The Telegraph Online	15/05/2022
Moodie, C.	Calories on menu will just drive us all Nandoolally	The Sun	12/04/2022
Moore, C.	The curse of childhood obesity begins at home	The Telegraph Online	24/06/2018
Morgan, E.	Obesity can't be tackled until we address the trauma that causes it	The Guardian	30/07/2020

Morrison, R.	Calorie counts on menus do work! Requiring restaurants to display nutritional information forces them to make their dishes healthier, researchers find	Mail Online	30/12/2021
Mosley, M.	Calories on menus won't slim us	Daily Mail	02/04/2022
Neville, S.; Scheherazade, D.	UK considers listing calories on restaurant menus	Financial Times (FT.Com)	24/06/2018
Ng, K.	Would more labelling really help us eat less sugar?	Independent Online	19/08/2021
Nield, L.; Paxman, J.	Why the new obesity plan won't work for everyone	The Independent	11/08/2020
O'Donoghue, P.	Diners 'eat less when calories are included on a menu'	thetimes.co.uk	20/09/2019
O'Flynn, P.	We must all do our bit in helping win the war on obesity	The Daily Express	27/07/2020
Odell, M.; Evans, H.	Calories on menus? Date night is ruined!	thetimes.co.uk	13/04/2022
Orbach, S.	Britain's obesity strategy ignores the science: dieting doesn't work	The Guardian	28/07/2020
Payne, M.	Loose Women's Nadia Sawalha confesses calorie counting led her to 'obesity' as she debates the government's new initiative	Mail Online	17/04/2022
Pearson-Jones, B.	Michelin-starred chefs lead backlash to calorie counts on menus as they are introduced in government scheme, saying it encourages eating disorders and will 'lead to boring, tick-box cooking'	Mail Online	02/04/2022
Petter, O.	Calorie counts on restaurant menus reduce how much people eat by 12%, study finds	Independent Online	27/02/2018
Pickles, K.	2-for-1 deals on junk food will be banned to cut obesity	Daily Mail	23/06/2018
Pickles, K.	Two-for-one deals on junk food will be banned in major obesity crackdown amid plans to end free soft drink refills	Mail Online	23/06/2018
Pickles, K.	Britain's diabetes time-bomb: Rise of Type 2 will cause heart attacks and strokes to soar over the coming years with 30% increase in serious illnesses linked to the condition	Mail Online	23/08/2018
Pickles, K.	Family doctors will be 'weight coaches' and sweets will be BANNED at checkouts as part of Boris Johnson's fight against obesity	Mail Online	27/07/2020

Pickles, K.	Coffee shops in the dock: Health chiefs warns high street chains' 'little nudges' to buy sweet treats are fuelling obesity crisis	Mail Online	15/09/2018
Pickover, E.	How menus in restaurants and cafes can tackle obesity	i	27/02/2018
Poulter, S.	The restaurant treats that take 8 hours to walk off	Daily Mail	12/09/2019
Roberts, E.	Why calorie counts on menus could actually be worse for our health	The Telegraph Online	06/04/2022
Shah, F.	Experts warn move to calorie-labelled menus is ‘problematic’	Independent Online	06/04/2022
Sheldrick, G.	Label food risks say experts as diabetes soars	The Daily Express	21/05/2018
Shipman, T.	Restaurants will be forced to display dishes' calorie content	The Sunday Times	27/05/2018
Shortall, E.	Calorie counts on menu study offers recipe to tackle obesity	The Sunday Times	03/03/2019
Simmons, E.	We don't need Ocado nagging us to cut the calories in our weekly shop	Mail Online	24/08/2019
Simmons, E.	Why putting calories on restaurant menus fills me with fear	Mail Online	08/08/2020
Sitwell, W.	Calorie-counting will kill our restaurants	The Daily Telegraph	29/07/2020
Smith, G.	Calorie counts turn dining out into a maths problem	i	19/10/2019
Smyth, C.	Restaurants urged to trim portions	thetimes.co.uk	13/12/2018
Smyth, C.	Ban on junk food adverts likely within two years	The Times	28/07/2020
Smyth, C.	Junk food ads banned online and before 9pm on television	The Times	12/05/2021
Smyth, C.; Coates, S.	Ban on junk food deals as obesity drive unites MPs	thetimes.co.uk	25/04/2018
Spencer, B.	Printing the calorie count of food and drinks on restaurant menus could reduce how much diners eat by 12%	Mail Online	27/02/2018
Spencer, B.; Blanchard, S.	Ministers push on with controversial plans to force ALL restaurants, cafes and even takeaways to put calorie labelling on menus as soon as next year despite widespread opposition	Mail Online	14/09/2018
Stern, C.	Trying to stay healthy? DON'T get duped by the menu! The seven sneaky things to avoid when dining out at a restaurant, from anything 'crispy' or 'creamy' to misleading SALADS	Mail Online	10/07/2018
Stoppard, M.	Energy drink ban for kids is the right move	The Daily Mirror	06/08/2018

Stoppard, M.	The labelling of calories on menus	The Daily Mirror	25/06/2021
Strimpel, Z.	Boris must control his urge to meddle with our diet	The Sunday Telegraph	02/08/2020
Stubley, P.	Restaurant meals and alcohol to get calorie labels in Boris Johnson’s obesity crackdown, reports say	Independent Online	25/07/2020
Swan, T. M.	Pub landlords: beer pump calorie counts are 'a creeping nanny state on steroids'	The Telegraph Online	15/04/2021
Swerling, G.	Scrap calorie counts on menus to cut risk of eating disorders, say MPs	The Telegraph Online	09/04/2021
Swinford, S.	Plan to force all restaurants, cafes and takeaways to display calorie counts on menus sparks Cabinet row	The Telegraph Online	05/09/2018
Swinford, S.; Lay, K.	Diet plans prescribed in Boris Johnson’s war on obesity	thetimes.co.uk	27/07/2020
Swinford, S.; Smyth, C.	Junk food adverts to be banned on TV before 9pm watershed	The Times	24/07/2020
Tapper, J.	Calories on menus ‘may not be helpful’ in drive against obesity	The Guardian	23/01/2022
Tapsfield, J.	Tough obesity crackdown could see ban on BOGOF deals for junk food and no TV adverts before 9pm after plans get cross-party backing	Mail Online	25/04/2018
Tapsfield, J.	Boris pushes ahead with 'nanny state' total BAN on online junk food ads after his own Covid battle - amid fears avocados, salmon, Marmite and HOUMOUS could fall foul of new rules	Mail Online	11/05/2021
The Daily Mail	3 in 4 want all restaurants to show calories	Scottish Daily Mail	09/03/2018
The Daily Mail	More than three-quarters of people want restaurants and takeaways to display calorie labelling on menus	Mail Online	09/03/2018
The Daily Telegraph	Fat chance	The Daily Telegraph	05/09/2018
The Daily Telegraph	'The bottom line is that you don't need to count calories'	The Daily Telegraph	05/09/2020
The Sun	The Sun Says: Weight a minute	The Sun	11/10/2017
The Sun	Calorie count plea	The Sun	09/03/2018
The Sun	Calories menu aid	The Sun	17/10/2019
The Sun	The Sun Says: Junk it, Boris	The Sun	25/07/2020

Sunday Times	We still need justice, not sabre-rattling, on Bloody Sunday	sundaytimes.co.uk	10/03/2019
The Telegraph	Letters: Archbishop of Canterbury wants higher taxes, but not for his Church	The Telegraph Online	07/09/2018
The Times	Food for Thought	The Times	17/10/2019
The Times	Counting the calories on restaurant menus	The Times	11/04/2022
The Times	Overcoming Obesity	The Times	19/05/2022
Uttley, H.	Struggling restaurants face fresh hammer blow from menu calorie rule	The Telegraph Online	28/07/2020
Vaughan, R.	Government's 'own goal' as Johnson takes on obesity time bomb	i	27/07/2020
Vincent, M.	The diet myths that EVERYONE falls victim to: Professor tells This Morning that you can skip breakfast and says calorie counting and exercise won't help you lose weight	Mail Online	27/08/2020
Walker, S.	Covid and the eating disorder crisis	The Telegraph Online	10/08/2020
Wallace, G.	Calorie info on menus... help to lose weight or too much nanny state?	The Sun	14/05/2021
Wallace, M.	A contradictory fat-fighting strategy	i	28/07/2020
Wardle, S.	Energy drinks face ban in child obesity battle	i	25/06/2018
Watson, S.	Want to lose weight? Turn the heating off and book a photographer	The Daily Telegraph	07/03/2018
Waugh, D.	Calorie counts on menus will not make any of us thinner	i	08/09/2018
Webber, E.	Conservatives struggle with their body image	thetimes.co.uk	27/07/2020
Wheeler, C.	Ministers mull ban on sale of energy drinks to children	sundaytimes.co.uk	24/06/2018
Wilcock, D.	'Sin tax' on sugary fizzy drinks could be extended to chocolates with adverts for sugary treats banned and health warnings slapped on alcohol bottles in anti-obesity plans being considered by Boris Johnson	Mail Online	28/07/2020
Wilson, A.	Why Boris Johnson’s obesity campaign could cause further suffering for those with eating disorders	Independent Online	16/09/2020
Wilson, B.	Spoon-Fed by Tim Spector review – food myths busted	The Guardian	05/08/2020
Wiseman, E.	Calorie counting doesn’t always add up	The Guardian	17/04/2022

Woodcock, A.	Boris Johnson once called health labels on wine bottles 'lunacy'	Independent Online	27/07/2020
Wooding, D.	Tiny cafes 'in calorie law let-off'	The Sun	21/04/2019
Wooller, S.	Calories on menu curbs fat	The Sun	27/02/2018
Wooller, S.	Bojo's battle of the bulge	The Sun	27/07/2020
Wooller, S.	Pubs to count calories to tackle obesity	Daily Mail	15/04/2021
Wooller, Shaun	Will putting meals' calorie counts on menu make you eat more healthily? Half of diners are likely to order more nutritious dishes when restaurants are forced to introduce the measure this week, study suggests	Mail Online	04/04/2022
Wright, Oliver	Treasury hits back over healthier menus	The Times	05/09/2018
Yeo, Giles	Everything you think you know about calories is bunkum! Obesity expert Dr Giles Yeo reveals the six pain-free ways to cut down on your intake	Mail Online	24/12/2018
Yorke, Harry	Small cafes fear falling foul of junk food advertising ban	The Daily Telegraph	13/05/2021
Young, Sarah	Diabetes UK says calorie labelling plans for restaurants must not be watered down	Independent Online	06/06/2019
