# Supplementary material for: Obesity and Lifestyle Drift: Framing Analysis of Calorie Menu Labelling in England in News Media
Source: Int J Health Policy Manag. 2025 Apr 28;14:8649. doi: 10.34172/ijhpm.8649 (PMC12257196; doi:10.34172/ijhpm.8649)
Supplement: Supplementary file 3 — Reflexivity Atatement. [file ijhpm-14-8649-s003.pdf]

Article title: Obesity and Lifestyle Drift: Framing Analysis of Calorie Menu Labelling in England in News Media

Journal name: International Journal of Health Policy and Management (IJHPM)

Authors' information: Nancy Karreman*, Michael Essman, Benjamin Hawkins, Jean Adams, Martin White

MRC Epidemiology Unit, University of Cambridge, Cambridge, UK

***Correspondence to:** Nancy Karreman; Email: nancy.karreman@mrc-epid.cam.ac.uk

Citation: Karreman N, Essman M, Hawkins B, Adams J, White M. Obesity and lifestyle drift: framing analysis of calorie menu labelling in England in news media. Int J Health Policy Manag. 2025;14:8649. doi:10.34172/ijhpm.8649

Supplementary file 3. Reflexivity Statement

This work was conducted by an interdisciplinary team of academic researchers with experience in framing and discourse analysis, policy evaluation, and dietary public health. JA was the lead investigator of a NIHR-commissioned evaluation of CL, on which MW was a co-investigator and ME was a researcher employed on the project (NIHR200689). As an author team, we embrace the importance of critically reflecting on our positioning as researchers, our beliefs, and our experience in relationship to the work presented in this study. We are all committed to a vision of equitable health improvement that includes some form of food-focussed government policy intervention alongside changes to other, structural sources of inequity. Our analysis necessarily reflects these personal and professional investments. Our reflexivity practice included a journal kept by lead author (NK) throughout the research process and co-writing of this section. During the analysis phase, NK also met regularly with the team most involved in analysis (JA, MW, BH) and sought alternative interpretations and views.
